# Pulmonary artery catheter use in adult patients undergoing cardiac surgery: a retrospective, cohort study

**DOI:** 10.1186/s13741-018-0103-x

**Published:** 2018-10-25

**Authors:** Andrew D. Shaw, Michael G. Mythen, Douglas Shook, David K. Hayashida, Xuan Zhang, Jeffrey R. Skaar, Sloka S. Iyengar, Sibyl H. Munson

**Affiliations:** 1 Department of Anesthesiology, Vanderbilt University Medical Center, Nashville, TN USA; 2 University College London Hospitals NIHR Biomedical Research Centre, London, UK; 3 Department of Anesthesiology, Brigham and Women’s Hospital, Boston, MA USA; 4 Boston Strategic Partners, Inc., Boston, MA USA; 5 Department of Anesthesiology and Pain Medicine, University of Alberta, 2-150 Clinical Sciences Building, Edmonton, AB T6G 2G3 Canada

**Keywords:** Cardiac surgery, Length-of-stay, Pulmonary artery catheter (PAC), Major morbidity, Mortality, Cardiopulmonary complications, Infectious complications

## Abstract

**Background:**

The utility of pulmonary artery catheters (PACs) and their measurements depend on a variety of factors including data interpretation and personnel training. This US multi-center, retrospective electronic health record (EHR) database analysis was performed to identify associations between PAC use in adult cardiac surgeries and effects on subsequent clinical outcomes.

**Methods:**

This cohort analysis utilized the Cerner Health Facts database to examine patients undergoing isolated coronary artery bypass graft (CABG), isolated valve surgery, aortic surgery, other complex non-valvular and multi-cardiac procedures, and/or heart transplant from January 1, 2011, to June 30, 2015. A total of 6844 adults in two cohorts, each with 3422 patients who underwent a qualifying cardiac procedure with or without the use of a PAC for monitoring purposes, were included. Patients were matched 1:1 using a propensity score based upon the date and type of surgery, hospital demographics, modified European System for Cardiac Operative Risk Evaluation (EuroSCORE II), and patient characteristics. Primary outcomes of 30-day in-hospital mortality, length of stay, cardiopulmonary morbidity, and infectious morbidity were analyzed after risk adjustment for acute physiology score.

**Results:**

There was no difference in the 30-day in-hospital mortality rate between treatment groups (OR, 1.17; 95% CI, 0.65–2.10; *p* = 0.516). PAC use was associated with a decreased length of stay (9.39 days without a PAC vs. 8.56 days with PAC; *p* < 0.001), a decreased cardiopulmonary morbidity (OR, 0.87; 95% CI, 0.79–0.96; *p* < 0.001), and an increased infectious morbidity (OR, 1.28; 95% CI, 1.10–1.49; *p* < 0.001).

**Conclusions:**

Use of a PAC during adult cardiac surgery is associated with decreased length of stay, reduced cardiopulmonary morbidity, and increased infectious morbidity but no increase in the 30-day in-hospital mortality. This suggests an overall potential benefit associated with PAC-based monitoring in this population.

**Trial registration:**

The study was registered at clinicaltrials.gov (NCT02964026) on November 15, 2016.

**Electronic supplementary material:**

The online version of this article (10.1186/s13741-018-0103-x) contains supplementary material, which is available to authorized users.

## Background

Pulmonary artery catheters (PACs) were introduced in 1970 (Swan et al. 1970) and have advantages over clinical assessment alone for predicting certain cardiac indices (Connors Jr et al. 1983; Iberti and Fisher 1983), detecting hemodynamic abnormalities (Hines 1990), and facilitating oxygen delivery-based protocols (Lobo et al. 2000; Boyd et al. 1993) that may decrease mortality during major surgery (Gurgel and do Nascimento Jr 2011). A subsequent retrospective study showed increased mortality and utilization of health care resources in critically ill intensive care unit (ICU) patients with PACs (Connors Jr et al. 1996), but other studies have shown either no harm or benefit associated with PAC use in critically ill (Harvey et al. 2005; Murdoch et al. 2000), high-risk, elderly, and surgical patients (Sandham et al. 2003), or in patients with symptomatic heart failure (Binanay et al. 2005).

Multiple studies have addressed PAC use in cardiac surgery patients, who currently receive 30% of PACs (Bernard et al. 2000). These range from increased mortality and a greater risk of severe organ complications associated with PAC use during coronary artery bypass grafting (CABG) (Schwann et al. 2011), increased mortality in high-risk cardiac surgery patients (Chiang et al. 2015), and no additional risk of cardiac arrest intraoperatively combined with a non-significant decrease in mortality and a lower likelihood of blood transfusion in patients undergoing CABG (Brovman et al. 2016). In non-emergent CABG patients, PAC use was associated with increased mortality, longer lengths of stay, and higher costs; however, these outcomes were more likely to be seen in hospitals with lower PAC use (Ramsey et al. 2000).

The lack of consensus regarding PAC-based monitoring outcomes in cardiac surgery has many potential reasons, including inadequate study design (Harvey et al. 2008), misinterpretation of data (Parviainen et al. 2006), non-standardized treatments, and training bias (i.e., regular PAC use may influence management of similar patients without PACs (Tuman 1997)). The accuracy and clinical utility of hemodynamic measurements obtained with a PAC correlate with proper catheter placement (Eisenberg et al. 1984) and correct data interpretation by physicians with PAC expertise (Iberti et al. 1994). Despite a lack of consensus and mixed messages surrounding PAC utility, PAC use increased significantly from 2010 to 2014 (Brovman et al. 2016).

Studies investigating clinical outcomes associated with PAC in the cardiac surgical population were performed exclusively in patients undergoing CABG procedures (Schwann et al. 2011; Ramsey et al. 2000), were performed using administrative data (Chiang et al. 2015; Brovman et al. 2016), had limited outcomes, or focused on factors associated with PAC utilization (Brovman et al. 2016). Hence, there is rationale for a study utilizing an electronic health record (EHR) database (including laboratory results and medications) to evaluate clinical outcomes associated with PAC use within all major cardiac procedures, with a cohort matched for hospital and patient characteristics inclusive of risk of mortality. In the current study, adult patients monitored with and without PAC in US hospitals performing a minimum of 100 qualifying cardiac surgeries from January 1, 2011, to June 30, 2015, were evaluated for primary outcomes of 30-day in-hospital mortality, major morbidity, and length of stay. We tested the hypothesis that PACs cause no harm and may provide some benefit for cardiac surgical patients.

## Methods

### Data source

The study was approved by the Vanderbilt University Institutional Review Board prior to data extraction. Given the retrospective nature of the study, the requirement for written informed consent was waived. HIPAA-compliant data were then extracted from the US Cerner Health Facts® (Cerner Corp., Kansas City, MO) database. In addition to hospital characteristics (bed size, teaching status, location) and encounter-level patient data (demographics, admission type, payer), comprehensive time-stamped medication orders, pharmacy records, laboratory results, admission and discharge diagnoses (International Classification of Diseases Ninth Revision Clinical Modification [ICD-9-CM codes]), and procedures were available. However, the database provided no information about the timing or duration of PAC insertion or physician rationale for PAC placement. The study was registered at clinicaltrials.gov (NCT02964026) in November 2016.

### Study population and exposure

Adult patients who underwent cardiac surgery between January 1, 2011, and June 30, 2015, were identified. Qualifying cardiac surgeries included isolated CABG, isolated valve, aortic, or other complex non-valvular surgery, multi-procedures, or heart transplant (see Additional file 1: Table S1 for cardiac procedures). If a patient received a percutaneous coronary intervention (PCI) prior to a CABG or valve procedure, they were excluded.

Cohorts were assigned based on PAC usage, and patients without a monitoring PAC comprised the control arm. Monitoring PACs were identified through ICD-9 codes (89.63 or 89.64) or Common Procedural Terminology-4 (CPT-4) code 93503 between the date of admission and the day following cardiac surgery, or via either of the following present on the date of cardiac procedure: ≥ 3 pulmonary artery pressure readings or a single pulmonary capillary wedge pressure reading. If a patient underwent multiple qualifying surgeries, the first in the database was utilized. Discrete encounters less than 4 h apart within the same hospital system were considered contiguous.

Exclusion criteria for this study were (1) patients < 18 years of age; (2) patients treated at hospitals that conduct fewer than 100 qualifying cardiac procedures per year (Table 1); (3) patients with missing records for demographics of age, gender, race, ICD-9 diagnosis, and procedure codes for the index visit or medications administered at the index visit; (4) no-PAC patients from an institution which does not have database documented use of monitoring PAC placement ICD-9 or CPT-4 codes; and (5) hospital length of stay < 48 h or > 180 days.

**Table 1:** Baseline patient characteristics for the matched cohort (3422 per arm)

Demographics	No PAC % (*n*)	PAC % (*n*)	Standardized difference*
Age at admission			
Mean (Std. Dev)	64.8 (11.5)	64.9 (11.8)	0.01
EuroScore II			
Mean (Std. Dev)	0.03 (0.03)	0.03 (0.03)	0.01
Gender			
Female	31.0 (1060)	32.1 (1099)	0.03
Male	69.0 (2362)	67.9 (2323)	
Race			
Black	6.5 (222)	7.3 (250)	0.03
White	86.2 (2951)	85.6 (2928)	
Asian	1.2 (41)	1.2 (40)	
Hispanic	0.6 (21)	0.6 (22)	
Other	5.5 (187)	5.3 (182)	
Institution bed size			
100–199	0.3 (10)	0.4 (15)	0.04
200–299	22.2 (761)	21.2 (727)	
300–499	16.0 (547)	15.1 (518)	
500+	61.5 (2104)	63.2 (2162)	
Admission type			
Elective	52.4 (1792)	53.3 (1823)	0.04
Non-elective	44.4 (1522)	44.2 (1511)	
Other/unspecified	3.2 (108)	2.6 (88)	
Payer			
Commercial	35.1 (1201)	35.2 (1204)	0.03
Government	48.3 (1653)	48.0 (1634)	
Payer not available	8.4 (288)	9.0 (307)	
Self-pay	8.2 (280)	8.1 (277)	
Teaching institution			
Yes	86.4 (2956)	86.3 (2953)	0.00
No	13.6 (466)	13.7 (469)	
US census region			
South	52.0 (1781)	53.0 (1815)	0.04
Northeast	22.9 (784)	21.6 (740)	
Midwest	14.3 (490)	13.9 (477)	
West	10.7 (367)	11.4 (390)	
Cardiac procedure class			
Isolated CABG	65.5 (2241)	64.0 (2191)	0.04
Isolated valve	19.8 (679)	21.1 (723)	
Multi-cardiac procedure	13.0 (446)	13.5 (461)	
Heart transplant	0.8 (26)	0.7 (23)	
Other complex non-valvular	0.8 (29)	0.7 (23)	
Aortic procedure	0.0 (1)	0.0 (1)	

*Standardized differences are reported as absolute values

### Outcomes

A combination of laboratory test values and medical codes were used to investigate outcomes. Primary outcomes were in-hospital mortality, cardiopulmonary morbidity composite, infectious morbidity composite, and index admission hospital length of stay (LOS). In-hospital mortality was determined for the first 30 days from index procedure date. Other primary and exploratory outcomes were determined from day 1 post-cardiac surgery through index discharge or through 30 days post-discharge (as noted in Additional file 1: Figures S4a–S4c; except Kidney Disease: Improving Global Outcomes Acute Kidney Injury (KDIGO AKI), which was determined through post-op day 10). The cardiopulmonary morbidity composite included dysrhythmia, new-onset heart failure, major adverse cardiac events (MACE) scores, cardiac complications, respiratory failure, use of ventilator, hemorrhage, and transfusion. This outcome did not include exploratory cardiovascular (CV) outcomes, whose definition included administration of inotropes and/or vasopressors, as these medications were received by the majority of the study population. Sequential organ failure assessment [SOFA] CV [includes inotropes; 68% of study cohort abnormal] and CV failure [receipt of inotropes and vasopressors; 84% of cohort] are not considered a complication. Data for SOFA CV are reported in Additional file 1: Table S5. The infectious morbidity composite included, in addition to diagnosed infection, confirmed pneumonia, bacteremia, urine infection, and catheter-associated blood stream infection, all of which included the requirement of a positive culture from the relevant sample type as well as a white blood cell count (WBCC) > 12 × 10^3^/μL on the same day to + 1 day of lab draw and antibiotic administered on the same day to + 3 days of lab draw (Table S2).

Exploratory outcomes included the following morbidity variables: AKI, gastrointestinal complication, liver complication, neurologic complication, SOFA CV (Vincent et al. 1996), unplanned readmissions, and all-cause readmissions. Additional file 1: Table S2 provides outcome definitions, and Additional file 1: Table S3 provides a comprehensive list of medical codes, lab results, and medications utilized for outcomes.

As medications and medical diagnosis and procedure codes were required in the EHR for study inclusion, all such patient outcomes were evaluable. For SOFA CV, mean arterial pressure (MAP) was not available for 7% of patients. All thresholds, parameters, and codes are further described in Additional file 1: Tables S2, S3. “Overall” or “composite” summary outcomes indicate that one or more conditions within the outcome were met. All listed exploratory outcomes met a minimum requirement of 80% data completeness, with the exception of serum creatinine (SCr) levels, which were missing in 33% of post-operative patients. Missing SCr was imputed as described in the Additional file 1: Information S1.

### Statistical analyses

To balance the two groups for risk of mortality, a modified European System for Cardiac Operative Risk Evaluation (EuroSCORE II) (Nashef et al. 2012) was calculated and utilized within the propensity matching algorithm. This variable was calculated as previously reported (Nashef et al. 2012), with minor modifications based on the data available within Cerner Health Facts® (Additional file 1: Information S2). Disease- and operation-related factors were defined using ICD-9 and CPT-4 codes, medications, and lab results.

The Acute Physiology Score (APS) portion of the Acute Physiology and Chronic Health Evaluation II (APACHE II) was assessed from the day of the index surgery through post-op day 1 using the worst value for each APS parameter prior to aggregation into the APS score. Missing values were imputed to the unmatched cohort study mean.

Patient demographics (age, gender, race, general type and year of cardiac procedure, and admission type and source) and institutional profiles (teaching status, bed size, US census region, urban/rural designation, acute care designation, and payer mix) were compared across the two exposure groups. The Elixhauser algorithm (Elixhauser et al. 1998) was used to identify and classify baseline comorbidities (using administrative and DRG codes via HealthCare Utilization Project, Agency for Healthcare Research and Quality software) (Comorbidity Software, Version 3.7 HCUP Comorbidity Software 2015). To maintain mutual exclusivity between comorbidities and outcomes, the ICD-9-CM diagnosis codes used to define complications were removed from the Elixhauser algorithm. The following ICD-9 codes were removed: 557.9 (peripheral vascular disease), 586 (unspecified renal failure), and all codes for valvular heart disease. Data were compared using *t* tests for continuous variables and chi-square tests for categorical variables. A two-sided *p* value of less than 0.05 denoted statistical significance.

Using a multiple logistic regression approach (with stepwise elimination for model selection), the probability for PAC exposure was estimated based on baseline demographics, year of cardiac procedure, hospital characteristics, and modified EuroSCORE II. Patients with similar propensity scores were matched 1:1 using greedy matching to reduce observable confounding (Parsons 2004). To assess match goodness of fit, baseline variables were compared between the unmatched and matched cohorts and standardized differences were calculated, for which a difference > 0.1 is considered unbalanced (Austin 2011). Outcomes were compared based on actual exposure to a monitoring PAC. The Elixhauser weight loss comorbidity, which remained unbalanced after propensity score-based matching (standardized difference = 0.48), and the APS portion of APACHE II were utilized to adjust all outcomes in the matched cohorts (Knaus et al. 1985). For binary outcomes, unadjusted and adjusted odds ratios and 95% confidence intervals (CI) are reported. Quantile (median) regression methods (Koenker 2013) were utilized to examine the effect of PAC use on hospital LOS, as the data were positively skewed. The Bonferroni correction was applied to the primary outcomes with significance level thus set at *p* < 0.0125 for each of the four outcomes. All analyses were performed using SAS® 9.4.

## Results

### Patient selection and cohort matching

**Fig. 1:**
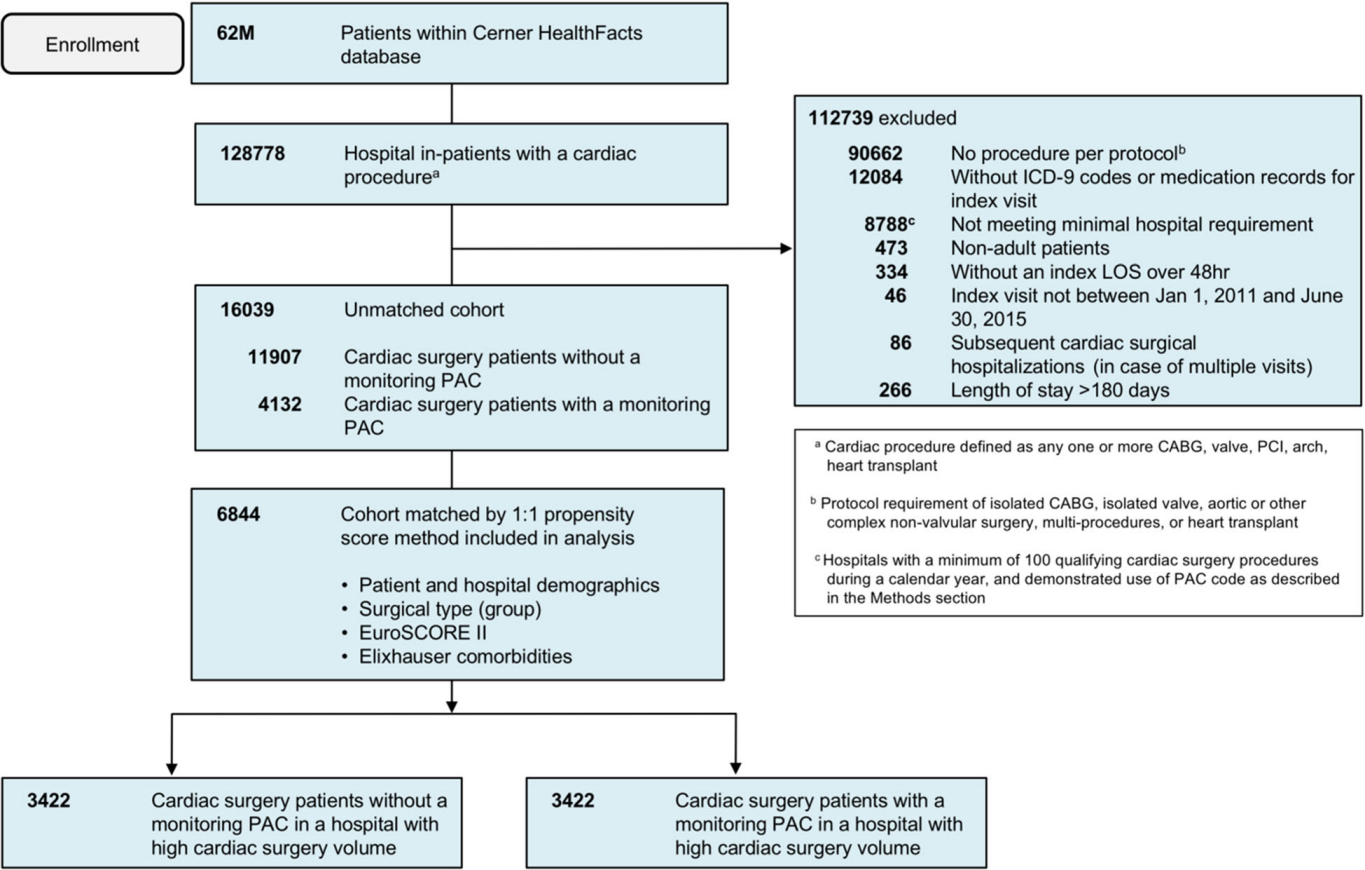
Patient selection and flow diagram

**Fig. 2:**
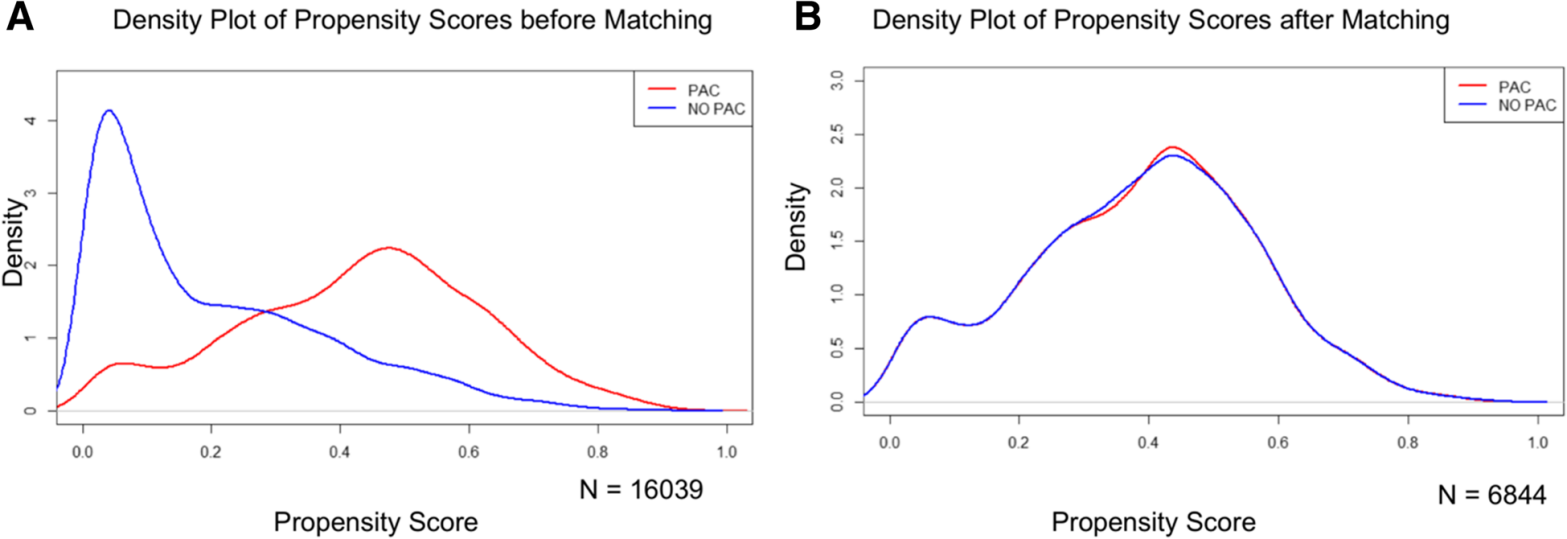
Propensity score matching of the study population. **a** Following the selection of patients with a qualifying cardiac procedure, patients were divided into two cohorts based on use (or non-use) of a PAC. **b** A propensity score-based match was performed with the PAC and no-PAC populations to generate matched cohorts of 3442 patients for analysis

Patients were included/excluded based on the criteria in Fig. 1. From 62 million in- and outpatients, 128,778 inpatients had a cardiac procedure. Following application of eligibility criteria, an unmatched study population of 16,039 patients remained. Propensity score matching was conducted based on patient and hospital demographics, surgery type, EuroSCORE II (Nashef et al. 2012), and patient comorbidities (Elixhauser et al. 1998), generating matched cohorts of 3422 patients each that received or did not receive a monitoring PAC (Fig. 2). The baseline characteristics between cohorts were not significantly different (Table 1). The majority of patients, 85.3%, underwent a CABG or valve procedure (< 1% received a heart transplant). EuroSCORE II values did not differ between arms, which are inclusive of variables such as procedure urgency, critical preoperative state (use of vasopressors immediately prior to surgery), left ventricular function, weight of intervention, procedures of the thoracic aorta, among other key characteristics (Supplemental Information S2). Comorbidities were characterized based on the Elixhauser comorbidity index (Elixhauser et al. 1998) (Table 2). Notably, the Elixhauser weight loss comorbidity remained unbalanced after propensity score-based matching (4.9% vs 3.9%, standardized difference = 0.48), and this parameter was included in the risk-adjusted outcome models.

**Table 2:** Elixhauser comorbidities for the matched cohort (3422 per arm)

Elixhauser parameters^&^	No PAC % (*n*)	PAC % (*n*)	Standardized difference*
Congestive heart failure	4.7 (162)	5.1 (175)	0.02
Circulatory disease	1.8 (62)	1.6 (54)	0.02
Peripheral vascular disease	16.0 (547)	14.5 (495)	0.04
Paralysis	1.8 (62)	1.8 (62)	0.00
Neurologic disease	5.1 (176)	4.8 (163)	0.02
Chronic lung/COPD	22.2 (759)	22.1 (756)	0.00
Renal failure	15.2 (521)	16.3 (559)	0.03
Diabetes	35.5 (1216)	34.8 (1191)	0.02
Diabetes with complications	6.4 (220)	6.4 (219)	0.00
Hypothyroidism	10.3 (351)	9.4 (323)	0.03
Liver disease	1.8 (60)	1.8 (60)	0.00
Peptic ulcer disease and bleeding	0.0 (0)	0.1 (2)	0.00
Acquired immune deficiency syndrome	0.1 (3)	0.1 (3)	0.00
Lymphoma	0.3 (11)	0.4 (14)	0.02
Metastatic cancer	0.4 (12)	0.2 (8)	0.02
Cancer	1.2 (41)	1.3 (43)	0.01
Rheumatoid arthritis	2.1 (71)	2.2 (76)	0.01
Coagulopathy	20.0 (685)	19.1 (652)	0.02
Obesity	21.8 (746)	22.6 (773)	0.02
Weight loss	4.9 (168)	3.9 (134)	0.48
Electrolyte disorder	33.0 (1128)	33.5 (1146)	0.01
Chronic blood loss anemia	1.6 (55)	1.5 (51)	0.01
Deficiency anemia	21.3 (728)	21.4 (733)	0.00
Alcohol use disorder	3.7 (125)	3.2 (109)	0.03
Drug dependence	2.0 (69)	2.1 (72)	0.01
Psychoses	2.1 (71)	2.7 (94)	0.04
Chronic depression	9.0 (308)	8.8 (301)	0.01
Complicated hypertension	76.6 (2622)	74.9 (2563)	0.04

^&^To maintain mutual exclusivity between comorbidities and outcomes, the ICD-9-CM diagnosis codes used to define complications were removed from the Elixhauser algorithms. The following ICD-9 codes were removed: 557.9 (peripheral vascular disease), 586 (unspecified renal failure), and all codes for valvular heart disease

*Standardized differences are reported as absolute values

### Primary adjusted and unadjusted outcomes

**Fig. 3:**
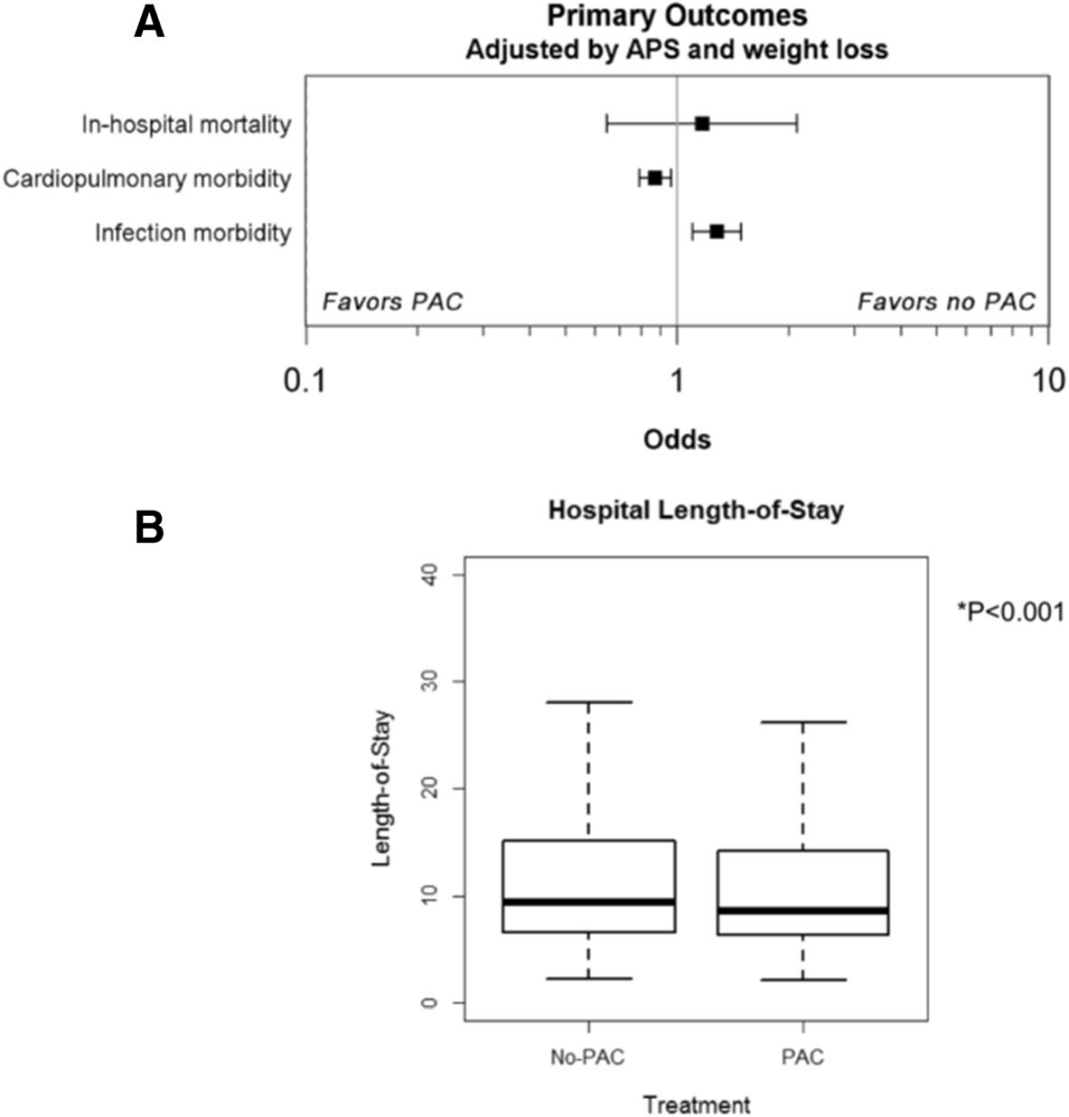
Primary outcomes associated with PAC use in cardiac surgery. **a** In-hospital mortality determined for the first 30 days from index procedure date, cardiopulmonary morbidity, infectious disease morbidity, and **b** length of stay for 6844 propensity score-matched pairs; the plot shows a median box plot with interquartile range (IQR) in the box and whiskers of 1.5 × IQR

The primary outcomes assessed were in-hospital mortality, a cardiopulmonary morbidity composite, an infectious disease morbidity composite, and hospital length of stay (Fig. 3) determined from day 1 post-cardiac surgery through hospital discharge or death. In-hospital 30-day mortality was not significantly different between patients with or without a monitoring PAC (OR, 1.17; 95% CI, 0.65–2.10; *p* = 0.516). Out of 3422 subjects, *n* = 22 in the no-PAC group (0.6%), and *n* = 24 in the PAC group (0.7%) expired. The cardiopulmonary morbidity composite showed significantly improved outcomes with PAC (OR, 0.87; 95% CI, 0.79–0.96; *p* < 0.001; no-PAC *n* = 1246 and PAC *n* = 1141). This was driven by new-onset heart failure (OR, 0.79; 95% CI, 0.68–0.93; *p* = 0.003), respiratory failure outcomes (OR, 0.62; 95% CI, 0.49–0.79; *p* < 0.001), and hemorrhage (OR, 0.62; 95% CI, 0.39–0.97; *p* = 0.038). Conversely, the infectious morbidity composite favored patients receiving no PAC (OR, 1.28; 95% CI, 1.10–1.49; *p* < 0.001; no-PAC *n* = 351 and PAC *n* = 429), as PAC patients displayed a significant increase in bacteremia (OR, 1.36; 95% CI, 1.02–1.82 *p* = 0.036) and urinary tract infection (OR, 1.58; 95% CI, 1.21–2.06; *p* < 0.001). The other components of each composite were not significantly different between the PAC and no-PAC populations (Additional file 1: Figure S4, Table S5).

Analysis of hospital length of stay revealed that PAC use was associated with a statistically significant decrease in length of stay when compared with patients without a PAC (median length of stay: PAC, 8.56 days vs no-PAC, 9.39 days; *p* < 0.001). Unadjusted values for all outcomes are shown in Additional file 1: Table S5. Unadjusted outcomes were similar to adjusted outcomes; in-hospital mortality was not significantly different between the groups (OR, 1.09; 95% CI, 0.61–1.95; *p* = 0.706). Unadjusted outcomes for cardiopulmonary and infectious morbidity composite outcomes were (OR, 0.87; 95% CI, 0.79–0.97; *p* < 0.001) and (OR, 1.25; 95% CI, 1.08–1.46; *p* < 0.001) respectively. Within individual components of these composite measures, the unadjusted values were similar and retained significance (Additional file 1: Table S5), except for the increased risk of bacteremia, which was not statistically significant (OR, 1.32; 95% CI, 0.99–1.75; *p* = 0.060).

### Exploratory outcomes

The matched PAC and no-PAC cohorts were also examined for a number of individual exploratory outcomes, including renal, gastrointestinal, liver, neurologic, and cardiac complications (Additional file 1: Figure S4, Table S5). Among these outcomes, adjusted (OR, 0.86; 95% CI, 0.75–0.97; *p* = 0.016) and unadjusted (OR, 0.85; 95% CI, 0.75–0.97; *p* = 0.012) post-operative KDIGO AKI were significantly decreased in patients who received a monitoring PAC. SOFA CV was found to be decreased in patients with a monitoring PAC when unadjusted values were evaluated (OR, 0.89; 95% CI, 0.80–0.99 *p* = 0.028), but was not significant with adjusted values (OR, 0.95; 95% CI, 0.85–1.06; *p* = 0.338). Neither unplanned readmissions nor all-cause readmissions showed statistically significant differences between the PAC or no-PAC groups (unplanned: OR, 1.06; 95% CI, 0.92–1.23; *p* = 0.418; all-cause: OR, 0.91; 95% CI, 0.82–1.00; *p* = 0.050; Additional file 1: Table S5). No significant differences were found in renal replacement therapy, transfusions, gastrointestinal complications, cholecystitis, and neurological outcomes.

## Discussion

Since Connors et al. initially questioned the utility of the pulmonary artery catheter (Connors Jr et al. 1996) in critically ill patients, numerous studies have investigated the effect of PACs in various patient populations using both prospective and retrospective study designs (Schwann et al. 2011; Harvey et al. 2005). These studies have all been influenced to varying degrees by a number of factors, including historical controls, database limitations, population size, population heterogeneity, and PAC expertise. By extracting data from a contemporary US EHR database of over 62 million patients, we sought to mitigate these factors and re-evaluate the potential harms and benefits of PAC use in a real-world cardiac surgery population incorporating laboratory tests and medications, particularly in light of recent work reporting increasing PAC use in cardiac surgeries (Brovman et al. 2016).

This large database population permitted the propensity score matching of patients based on patient and hospital characteristics, including risk of mortality which incorporated lab results, medications, and vital signs (via EuroSCORE II) and the exclusion of patients treated at hospitals performing fewer than 100 qualifying cardiac procedures per year and from institutions without documented use of monitoring PACs to account for the impact of PAC familiarity and expertise on outcomes. We believe that observational studies, such as this study, that are designed to look for evidence of harm (as opposed to benefits) are more robust to indication and selection bias, because health care providers do not make treatment choices with the intent of causing harm. As might be expected based upon current guidelines (Practice guidelines for pulmonary artery catheterization 2003), the unmatched populations did reveal that PACs were preferentially administered to patients with a higher risk profile (median modified EuroSCORE II: PAC 0.016, no-PAC 0.015; *p* = 0.004).

Using the propensity score-matched cohorts of cardiac surgery patients with or without a PAC, we found that PAC use was not associated with increased risk of harm as measured by in-hospital mortality (30 days). However, despite the large size of our study (6844 patients), because of the low mortality rate in both arms (0.6% no-PAC group; 0.7% PAC group), our study did not have sufficient power to detect a definitive outcome (Pearson chi-square; power 0.0575 at *α* = 0.0125). PAC use was associated with a statistically significant decrease in length of hospital stay and a significant decrease in the cardiopulmonary morbidity composite, suggesting potential benefits associated with PAC monitoring. Our study also supports a previous study that reported a reduction in transfusions in PAC patients (Cohen et al. 2005). Although we did not observe a significant decrease in transfusion rates in the PAC population, we did find a significant decrease in hemorrhage, suggesting that PAC use may have a positive association with these two related outcome measures (it is important to note that the number of units transfused could not be evaluated within the database between the study arms).

Other retrospective analyses of PACs in cardiac surgeries have revealed conflicting results when assessing mortality. Earlier evaluations with small patient numbers (i.e., < 100) (Larson and Kyff 1989) found no impact, while studies with historical controls reported some benefit associated with PAC use (Schwann et al. 2002). More recently, two large, controlled, retrospective analyses of different national administrative databases (i.e., the National Inpatient Sample and the National Anesthesia Outcomes Registry) reached different conclusions on PAC use and mortality risk, with one study finding a significant increase in mortality risk with PAC use (Chiang et al. 2015) and the other reporting a non-significant decrease in mortality risk with PAC use (Brovman et al. 2016). Notably, the former study (Chiang et al. 2015) found higher rates of mortality in high-risk patient groups such as octogenarian patients, and those with congestive heart failure. Similar to other studies (Binanay et al. 2005; Elliott et al. 1979), we found that PAC use was associated with urinary tract infections (UTIs) and bacteremia. Insertion of a PAC has been shown to be associated with infection (Mermel et al. 1991), but the nature of the observed increase in infection in our study population is unclear. We did not observe differences in line infection between the groups; therefore, it is more likely that the increase in bacteremia in patients monitored with a PAC is due to the higher UTI rate, perhaps due to indwelling Foley catheters which are not typically removed if a PAC is still present. Unfortunately, a limitation of our study is the lack of information about the timing of bladder catheterization (presumably immediately after anesthesia induction), total surgery time, and total bladder catheter dwell time. As placement of central venous catheters (CVCs) may not be routinely coded, examining an association between CVCs and infection is difficult. Another minor limitation is that EuroSCORE II was not designed for heart transplant patients; however, in the current study, patients were matched on cardiac procedure type, and heart transplant patients comprised a small proportion of the population that was not significantly different between arms (no-PAC: 0.8%; PAC 0.7%; *p* = 0.669).

Proper use and interpretation of PAC-derived data requires expertise and knowledge acquired during training and thereafter supplemented by frequent use. There is evidence to suggest that initial training in PAC use is important for high-quality outcomes (Practice guidelines for pulmonary artery catheterization 2003). Notably, in contrast to the current study, the sources for prior studies do not allow for propensity matching based on hospital demographics or the selection for facilities with documented PAC use to account for provider expertise and experience. This study performed a propensity match which included patient demographics inclusive of pre-existing conditions as Elixhauser comorbidities (e.g., pulmonary hypertension) as well as via the modified EuroSCORE II (inclusive of “critical preoperative state”; Additional file 1 Information S2). However, despite attempts to select for PAC expertise through selection of high volume cardiac surgery hospitals with PAC use, this analysis of EHRs does not indicate the level of experience of the PAC provider, whether the PAC was correctly used, or if a patient’s treatment was informed by PAC readings, highlighting additional study limitations. Local hospital policies and economic considerations influence PAC use (Ranucci 2006), and lack of information about these variables is an additional limitation of our study. A further difference between the current and prior database analyses is our ability to use both coding and clinical parameters to capture outcome measures and better match study arms. In the current analysis, outcomes such as KDIGO AKI and infectious disease are defined by both coding and laboratory test values (and for infectious disease, medications) rather than isolated claims data. EuroSCORE II calculation includes use of laboratory, medication, and vital signs in addition to medical diagnosis and procedure codes. The timing surrounding some of the cardiac components (those defined by medical diagnosis codes) of the cardiopulmonary outcome composite is unknown. Therefore, for example, a new onset of heart failure that occurs in the early phase such as during PAC insertion, or in the late phase of post-op cardiac surgery, cannot be discerned within this study. The database did not allow reliable analysis of CVC utilization within the study cohort, and therefore, this remains a limitation.

Recent years have seen considerable commercial support for new, non-invasive hemodynamic monitoring technologies proposed as PAC alternatives. However, despite reports suggesting neutral or negative outcomes associated with PAC use, and the development of competing monitoring technologies, the use of the PACs in cardiac surgery has remained robust—and perhaps has even increased (Brovman et al. 2016; Judge et al. 2015). This study was performed to investigate this apparent paradox through analysis of patients undergoing a full range of major cardiac procedures using lab values, medications, and vital signs available within an EHR database to both match and derive clinical outcomes. We found that the PAC cohort demonstrated significantly decreased length of stay and cardiopulmonary morbidity versus the no-PAC cohort. These improvements, in the absence of significant changes in in-hospital mortality risk, may help explain the recently reported increase in PAC use in cardiac surgical patients (Brovman et al. 2016; Judge et al. 2015).

## Conclusions

In this EHR database study of 6844 patients, we tested the hypothesis that PACs cause no harm and may provide some benefit in cardiac surgeries. We found that use of a PAC in adult cardiac surgery patients was associated with no increased risk of mortality (although insufficiently powered at the low observed mortality rates), a reduced length of stay, and reduced cardiopulmonary morbidity. However, PAC use was also associated with greater incidences of bacteremia and urinary infections, although not line infections. Overall, the lack of serious harm and potential for benefit associated with PAC-based monitoring should inform future prospective trials in well-defined patient populations (such as within those of high vs. low risk) in which patient outcomes for this familiar technology may be tested.

## Additional file

Additional file 1: **Table S1.** Qualifying cardiac surgical procedures and medical codes. **Information S1.** Detailed description of outcomes. **Table S2.** Outcomes definitions and criteria. **Table S3.** Summary of medical codes utilized within outcomes definitions. **Figure S4**a. Cardiopulmonary disease outcomes. **Figure S4**b. Infectious morbidity outcomes. **Figure S4**c. Other exploratory outcomes. **Table S5**a. Primary outcomes frequencies and statistics. **Table S5**b. Cardiopulmonary disease frequencies and models statistics. **Table S5**c. Infectious disease outcomes frequencies and models statistics. **Table S5**d. Other exploratory outcomes frequency and models statistics. **Information S2.** EuroSCORE II evaluation. **Table S6**. EuroSCORE II calculation. (DOCX 4625 kb)

## Abbreviations

APACHE II: Acute Physiology and Chronic Health Evaluation II
APS: Acute Physiology Score
CABG: Coronary artery bypass graft
CI: Confidence interval
CO: Cardiac output
CPT-4: Common Procedural Terminology-4
CV: Cardiovascular
CVC: Central venous catheter
EHR: Electronic health record
EuroSCORE II: European System for Cardiac Operative Risk Evaluation
ICD-9-CM: International Classification of Diseases Ninth Revision Clinical Modification
ICU: Intensive care unit
KDIGO AKI: Kidney Disease: Improving Global Outcomes Acute Kidney Injury
LOS: Length of stay
MACE: Major adverse cardiac events
MAP: Mean arterial pressure
OR: Odds ratio
PAC: Pulmonary artery catheter
PCI: Percutaneous coronary intervention
SCr: Serum creatinine
SOFA CV: Sequential organ failure assessment; cardiovascular
SOFA: Sequential organ failure assessment
UTI: Urinary tract infection
