# Supplementary material for: Pulmonary artery catheter use in adult patients undergoing cardiac surgery: a retrospective, cohort study
Source: Perioper Med (Lond). 2018 Oct 25;7:24. doi: 10.1186/s13741-018-0103-x (PMC6201566; doi:10.1186/s13741-018-0103-x)
Supplement: Supplementary file 1 — Table S1. Qualifying cardiac surgical procedures and medical codes. Information S1. Detailed description of outcomes. Table S2. Outcomes definitions and criteria. Table S3. Summary of medical codes utilized within outcomes definitions. Figure S4a. Cardiopulmonary disease outcomes. Figure S4b. Infectious morbidity outcomes. Figure S4c. Other exploratory outcomes. Table S5a. Primary outcomes frequencies and statistics. Table S5b. Cardiopulmonary disease frequencies and models statistics. Table S5c. Infectious disease outcomes frequencies and models statistics. Table S5d. Other exploratory outcomes frequency and models statistics. Information S2. EuroSCORE II evaluation. Table S6. EuroSCORE II calculation. (DOCX 4625 kb) [file 13741_2018_103_MOESM1_ESM.docx]

**ADDITIONAL FILE 1**

**Additional information Page number**

Table S1: Qualifying cardiac surgical procedures and medical codes 2

Information S1: Detailed description of outcomes 7

Table S2: Outcomes definitions and criteria 8

Table S3: Summary of medical codes utilized within outcomes definitions 9

Figure S4a: Cardiopulmonary disease outcomes 13

Figure S4b: Infectious morbidity outcomes 14

Figure S4c: Other exploratory outcomes 15

Table S5a: Primary outcomes frequencies and statistics 16

Table S5b: Cardiopulmonary disease frequencies and models statistics 16

Table S5c: Infectious disease outcomes frequencies and models statistics 16

Table S5d: Other exploratory outcomes frequency and models statistics 17

Information S2: EuroSCORE II evaluation 18

Table S6: EuroSCORE II calculation 20

References 22

**Table S1.** Qualifying cardiac surgical procedures and medical codes

| **ICD-9 or**  **CPT Code** | **Cardiac Procedure Description** |
| --- | --- |

**Coronary artery bypass graft (CABG) procedures**

| 36.1 | Bypass Anastomosis for Heart Revascularization |
| --- | --- |
| 36.10 | Aortocoronary Bypass for Heart Revascularization, Not Otherwise Specified |
| 36.11 | (Aorto)coronary Bypass of One Coronary Artery |
| 36.12 | (Aorto)coronary Bypass of Two Coronary Arteries |
| 36.13 | (Aorto)coronary Bypass of Three Coronary Arteries |
| 36.14 | (Aorto)coronary Bypass of Four or More Coronary Arteries |
| 36.15 | Single Internal Mammary-Coronary Artery Bypass |
| 36.16 | Double Internal Mammary-Coronary Artery Bypass |
| 36.17 | Abdominal - Coronary Artery Bypass |
| 36.19 | Other Bypass Anastomosis for Heart Revascularization |
| 33510 | Coronary artery bypass, vein only; single coronary venous graft |
| 33511 | Coronary artery bypass, vein only; 2 coronary venous grafts |
| 33512 | Coronary artery bypass, vein only; 3 coronary venous grafts |
| 33513 | Coronary artery bypass, vein only; 4 coronary venous grafts |
| 33514 | Coronary artery bypass, vein only; 5 coronary venous grafts |
| 33516 | Coronary artery bypass, vein only; 6 or more coronary venous grafts |
| 33517 | Coronary artery bypass, using venous graft(s) and arterial graft(s); single vein graft |
| 33518 | Coronary artery bypass, using venous graft(s) and arterial graft(s); 2 venous grafts |
| 33519 | Coronary artery bypass, using venous graft(s) and arterial graft(s); 3 venous grafts |
| 33521 | Coronary artery bypass, using venous graft(s) and arterial graft(s); 4 venous grafts |
| 33522 | Coronary artery bypass, using venous graft(s) and arterial graft(s); 5 venous grafts |
| 33523 | Coronary artery bypass, using venous graft(s) and arterial graft(s); 6 or more venous grafts |
| 33533 | Coronary artery bypass, using arterial graft(s); single arterial graft |
| 33534 | Coronary artery bypass, using arterial graft(s); 2 coronary arterial grafts |
| 33535 | Coronary artery bypass, using arterial graft(s); 3 coronary arterial grafts |
| 33536 | Coronary artery bypass, using arterial graft(s); 4 or more coronary arterial grafts |
| 33572 | Coronary endarterectomy, open, any method, of left anterior descending, circumflex, or right coronary artery performed in conjunction with coronary artery bypass graft procedure, each vessel |

**Valve procedures**

| 35 | Operations on Valves and Septa of heart | |
| --- | --- | --- |
| 35.0 | Closed Heart Valvotomy or Transcatheter Replacement of Heart Valve | |
| 35.00* | Closed Heart Valvotomy, Unspecified Valve | |
| 35.01* | Closed Heart Valvotomy, Aortic Valve | |
| 35.02* | Closed Heart Valvotomy, Mitral Valve | |
| 35.03* | Closed Heart Valvotomy, Pulmonary Valve | |
| 35.04* | Closed Heart Valvotomy, Tricuspid Valve | |
| 35.05 | Endovascular Replacement of Aortic Valve | |
| 35.06 | Transapical Replacement of Aortic Valve | |
| 35.07 | Endovascular Replacement of Pulmonary Valve | |
| 35.08 | Transapical Replacement of Pulmonary Valve | |
| 35.09 | Endovascular Replacement of Unspecified Heart Valve | |
| 35.1 | Open Heart Valvuloplasty without Replacement | |
| 35.10 | Open Heart Valvuloplasty without Replacement, Unspecified Valve | |
| 35.11 | Open Heart Valvuloplasty of Aortic Valve without Replacement | |
| 35.12 | Open Heart Valvuloplasty of Mitral Valve without Replacement | |
| 35.13 | Open Heart Valvuloplasty of Pulmonary Valve without Replacement | |
| 35.14 | Open Heart Valvuloplasty of Tricuspid Valve without Replacement | |
| 35.2 | Open and Other Replacement of Heart Valve | |
| 35.20 | Open and Other Replacement of Unspecified Heart Valve | |
| 35.21 | Open and Other Replacement of Aortic Valve with Tissue Graft | |
| 35.22 | Open and Other Replacement of Aortic Valve | |
| 35.23 | Open and Other Replacement of Mitral Valve with Tissue Graft | |
| 35.24 | Open and Other Replacement of Mitral Valve | |
| 35.25 | Open and Other Replacement of Pulmonary Valve with Tissue Graft | |
| 35.26 | Open and Other Replacement of Pulmonary Valve | |
| 35.27 | Open and Other Replacement of Tricuspid Valve with Tissue Graft | |
| 35.28 | Open and Other Replacement of Tricuspid Valve | |
| 35.3 | Operations on Structures Adjacent to Heart Valves | |
| 35.31 | Operations on Papillary Muscle | |
| 35.32 | Operations on Chordae Tendineae | |
| 35.33 | Annuloplasty | |
| 35.97 | Percutaneous Mitral Valve Repair with Implant | |
| 35.99 | Other Operations on Valves of Heart | |
| 33361 | Transcatheter aortic valve replacement (TAVR/TAVI) with prosthetic valve; percutaneous femoral artery approach | |
| 33362 | Transcatheter aortic valve replacement (TAVR/TAVI) with prosthetic valve; open femoral artery approach | |
| 33363 | Transcatheter aortic valve replacement (TAVR/TAVI) with prosthetic valve; open axillary artery approach | |
| 33364 | Transcatheter aortic valve replacement (TAVR/TAVI) with prosthetic valve; open iliac artery approach | |
| 33365 | Transcatheter aortic valve replacement (TAVR/TAVI) with prosthetic valve; transaortic approach (eg, median sternotomy, mediastinotomy) | |
| 33366 | Transcatheter aortic valve replacement (TAVR/TAVI) with prosthetic valve; transapical exposure (eg, left thoracotomy) | |
| 33367 | Transcatheter aortic valve replacement (TAVR/TAVI) with prosthetic valve; cardiopulmonary bypass support with percutaneous peripheral arterial and venous cannulation (eg, femoral vessels) | |
| 33368 | Transcatheter aortic valve replacement (TAVR/TAVI) with prosthetic valve; cardiopulmonary bypass support with open peripheral arterial and venous cannulation (eg, femoral, iliac, axillary vessels) | |
| 33369 | Transcatheter aortic valve replacement (TAVR/TAVI) with prosthetic valve; cardiopulmonary bypass support with central arterial and venous cannulation (eg, aorta, right atrium, pulmonary artery) | |
| 33405 | Replacement, aortic valve, with cardiopulmonary bypass; with prosthetic valve, other than homograft or stentless valve | |
| 33406 | Replacement, aortic valve, with cardiopulmonary bypass; with allograft valve (freehand) | |
| 33410 | Replacement, aortic valve, with cardiopulmonary bypass; with stentless tissue valve | |
| 33411 | Replacement, aortic valve; with aortic annulus enlargement, noncoronary sinus | |
| 33412 | Replacement, aortic valve; with transventricular aortic annulus enlargement (Konno procedure) | |
| 33413 | | Replacement, aortic valve; by translocation of autologous pulmonary valve with allograft replacement of pulmonary valve (Ross procedure) |
| 33415 | Resection or incision of subvalvular tissue for discrete subvalvular aortic stenosis | |
| 33422 | Valvotomy, mitral valve; open heart, with cardiopulmonary bypass | |
| 33430 | Replacement, mitral valve, with cardiopulmonary bypass | |
| 33460 | Valvectomy, tricuspid valve, with cardiopulmonary bypass | |
| 33465 | Replacement, tricuspid valve, with cardiopulmonary bypass | |
| 33468 | Tricuspid valve repositioning and plication for Ebstein anomaly | |
| 33472 | Valvotomy, pulmonary valve, open heart; with inflow occlusion | |
| 33474 | Valvotomy, pulmonary valve, open heart, with cardiopulmonary bypass | |
| 33475 | Replacement, pulmonary valve | |
| 33496* | Repair of non-structural prosthetic valve dysfunction with cardiopulmonary bypass (separate procedure) | |
| 33403 | Valvuloplasty, aortic valve; using transventricular dilation, with cardiopulmonary bypass | |
| 33425 | Valvuloplasty, mitral valve, with cardiopulmonary bypass | |
| 33426 | Valvuloplasty, mitral valve, with cardiopulmonary bypass; with prosthetic ring | |
| 33427 | Valvuloplasty, mitral valve, with cardiopulmonary bypass; radical reconstruction, with or without ring | |

***** Indicates procedures which required a code to indicate that the procedure was performed with cardiopulmonary bypass (CPB) – see below

**Codes used to verify use of Cardio-Pulmonary Bypass, when required, for certain valve procedures**

| 39.61 | Extracorporeal Circulation Auxiliary to Open Heart Surgery | | | |
| --- | --- | --- | --- | --- |
| 33405 | Replacement, aortic valve, with cardiopulmonary bypass; with prosthetic valve, other than homograft or stentless valve | | | |
| 33406 | Replacement, aortic valve, with cardiopulmonary bypass; with allograft valve (freehand) | | | |
| 33410 | Replacement, aortic valve, with cardiopulmonary bypass; with stentless tissue valve | | | |
| 33422 | Valvotomy, mitral valve; open heart, with cardiopulmonary bypass | | | |
| 33430 | Replacement, mitral valve, with cardiopulmonary bypass | | | |
| 33460 | Valvectomy, tricuspid valve, with cardiopulmonary bypass | | | |
| 33465 | Replacement, tricuspid valve, with cardiopulmonary bypass | | | |
| 33474 | Valvotomy, pulmonary valve, open heart, with cardiopulmonary bypass | | | |
| 33403 | Valvuloplasty, aortic valve; using transventricular dilation, with cardiopulmonary bypass | | | |
| 33425 | Valvuloplasty, mitral valve, with cardiopulmonary bypass; | | | |
| 33426 | Valvuloplasty, mitral valve, with cardiopulmonary bypass; with prosthetic ring | | | |
| 33427 | Valvuloplasty, mitral valve, with cardiopulmonary bypass; radical reconstruction, with or without ring | | | |
| 33120 | Excision of intracardiac tumor, resection with cardiopulmonary bypass | | | |
| 33305 | Repair of cardiac wound; with cardiopulmonary bypass | | | |
| 33315 | Cardiotomy, exploratory (includes removal of foreign body, atrial or ventricular thrombus); with cardiopulmonary bypass | | | |
| 33322 | Suture repair of aorta or great vessels; with cardiopulmonary bypass | | | |
| 33335 | Insertion of graft, aorta or great vessels; with cardiopulmonary bypass | | | |
| 33500 | Repair of coronary arteriovenous or arteriocardiac chamber fistula; with cardiopulmonary bypass | | | |
| 33504 | Repair of anomalous coronary artery from pulmonary artery origin; by graft, with cardiopulmonary bypass | | | |
| 33641 | Repair atrial septal defect, secundum, with cardiopulmonary bypass, with or without patch | | | |
| 33702 | Repair sinus of Valsalva fistula, with cardiopulmonary bypass; | | | |
| 33710 | Repair sinus of Valsalva fistula, with cardiopulmonary bypass; with repair of ventricular septal defect | | | |
| 33720 | | Repair sinus of Valsalva aneurysm, with cardiopulmonary bypass | |  |
| 33736 | | Atrial septectomy or septostomy; open heart with cardiopulmonary bypass | |  |
| 33814 | | Obliteration of aortopulmonary septal defect; with cardiopulmonary bypass | |  |
| 33853 | | | Repair of hypoplastic or interrupted aortic arch using autogenous or prosthetic material; with cardiopulmonary bypass | |
| 33860 | | | Ascending aorta graft, with cardiopulmonary bypass, includes valve suspension, when performed | |
| 33864 | | | Ascending aorta graft, with cardiopulmonary bypass with valve suspension, with coronary reconstruction and valve-sparing aortic root remodeling (eg, David Procedure, Yacoub Procedure) | |
| 33870 | | | Transverse arch graft, with cardiopulmonary bypass | |
| 33910 | | | Pulmonary artery embolectomy; with cardiopulmonary bypass | |
| 33916 | | | Pulmonary endarterectomy, with or without embolectomy, with cardiopulmonary bypass | |
| 33922 | | | Transection of pulmonary artery with cardiopulmonary bypass | |
| 33926 | | | Repair of pulmonary artery arborization anomalies by unifocalization; with cardiopulmonary bypass | |
| 33960 | | | Prolonged extracorporeal circulation for cardiopulmonary insufficiency; initial day | |
| 33961 | | | Prolonged extracorporeal circulation for cardiopulmonary insufficiency; each subsequent day | |
| 36822 | | | Insertion of cannula(s) for prolonged extracorporeal circulation for cardiopulmonary insufficiency (ECMO) | |

**Aortic procedures**

| 33852 | Repair of hypoplastic or interrupted aortic arch using autogenous or prosthetic material; without cardiopulmonary bypass | |
| --- | --- | --- |
| 33853 | Repair of hypoplastic or interrupted aortic arch using autogenous or prosthetic material; with cardiopulmonary bypass | |
| 33860 | Ascending aorta graft, with cardiopulmonary bypass, includes valve suspension, when performed | |
| 33864 | Ascending aorta graft, with cardiopulmonary bypass with valve suspension, with coronary reconstruction and valve-sparing aortic root remodeling (eg, David Procedure, Yacoub Procedure) | |
| 33870 | Transverse arch graft, with cardiopulmonary bypass |  |

**Other – Complex Nonvalvular procedures**

| 33120 | Excision of intracardiac tumor, resection with cardiopulmonary bypass |
| --- | --- |
| 33305 | Repair of cardiac wound; with cardiopulmonary bypass |
| 33315 | Cardiotomy, exploratory (includes removal of foreign body, atrial or ventricular thrombus); with cardiopulmonary bypass |
| 33322 | Suture repair of aorta or great vessels; with cardiopulmonary bypass |
| 33335 | Insertion of graft, aorta or great vessels; with cardiopulmonary bypass |
| 33500 | Repair of coronary arteriovenous or arteriocardiac chamber fistula; with cardiopulmonary bypass |
| 33504 | Repair of anomalous coronary artery from pulmonary artery origin; by graft, with cardiopulmonary bypass |
| 33641 | Repair atrial septal defect, secundum, with cardiopulmonary bypass, with or without patch |
| 33702 | Repair sinus of Valsalva fistula, with cardiopulmonary bypass |
| 33710 | Repair sinus of Valsalva fistula, with cardiopulmonary bypass; with repair of ventricular septal defect |
| 33720 | Repair sinus of Valsalva aneurysm, with cardiopulmonary bypass |
| 33736 | Atrial septectomy or septostomy; open heart with cardiopulmonary bypass |
| 33814 | Obliteration of aortopulmonary septal defect; with cardiopulmonary bypass |
| 33910 | Pulmonary artery embolectomy; with cardiopulmonary bypass |
| 33916 | Pulmonary endarterectomy, with or without embolectomy, with cardiopulmonary bypass |
| 33922 | Transection of pulmonary artery with cardiopulmonary bypass |
| 33926 | Repair of pulmonary artery arborization anomalies by unifocalization; with cardiopulmonary bypass |
| 35.61 | Repair of atrial septal defect with tissue graft |
| 35.51 | Repair of Atrial and Ventricular Septa with Prosthesis: Repair of Atrial Septal Defect with Prosthesis, Open Technique |
| 35.52 | Repair of Atrial and Ventricular Septa with Prosthesis: Repair of Atrial Septal Defect with Prosthesis, Closed Technique |
| 35.71 | Other and unspecified repair of atrial septal defect; foramen ovale |
| 38.05 | Embolectomy of plumonary artery |

**Heart transplant procedures**

| 33935 | Heart-lung transplant with recipient cardiectomy-pneumonectomy | |
| --- | --- | --- |
| 37.51 | Heart Transplantation |  |

**PCI procedures utilized as exclusion criteria if performed on the same day as a qualifying CABG or valve procedure**

| 36.01 | Single Vessel Percutaneous Transluminal Coronary Angioplasty or Coronary Atherectomy Without Mention of Thrombolytic Agent |
| --- | --- |
| 36.02 | Single Vessel Percutaneous Transluminal Coronary Angioplasty or Coronary Atherectomy With Mention of Thrombolytic Agent |
| 36.03 | Open Chest Coronary Artery Angioplasty |
| 36.04 | Intracoronary Artery Thrombolytic Infusion |
| 36.05 | Multiple Vessel Percutaneous Transluminal Coronary Angioplasty or Coronary Atherectomy Performed During the Same Operation, With or Without Mention Of Thrombolytic Agent |
| 36.06 | Insertion of Non-Drug-Eluting Coronary Artery Stent(s) |
| 36.07 | Insertion of Drug-Eluting Coronary Artery Stent(s) |
| 36.09 | Other Specified Removal of Coronary Artery Obstruction |

**Information S1:** Detailed description of outcomes

Exploratory outcomes included the following morbidity variables: Cardiovascular: Dysrhythmia, Major Adverse Cardiac Events (MACE) components of 1) post-operative stroke or cerebrovascular accident (Experienced Stroke); 2) post-operative revascularization or PCI (Underwent PCI); and 3) post-operative myocardial infarction (MI; Experienced MI)], cardiac complications [coded], new onset heart failure), Sequential Organ Failure Assessment (SOFA) CV [1] Abnormal [stages 1 – 4; includes blood pressure, Mean Arterial Pressure [MAP], vasoactive medication orders)]; respiratory (on ventilator >96 hours, on ventilator <96 hours, and respiratory failure [all coded]); infectious disease (infectious complications [ICD-9 codes], confirmed pneumonia [bronchoalveolar lavage or tracheal sample], bacteremia, urine Infection, or line Infection); bleeding (transfusion and hemorrhage [both via ICD-9 codes]); Kidney/Acute Kidney Injury (AKI; via renal failure and renal replacement therapy [both coded] and post-operative Kidney Disease Improving Global Outcomes [KDIGO] AKI); gastrointestinal complication (coded); neurologic complication (coded); and liver complication (Cholecystitis [coded]). See Supplemental Table 2 for outcome definitions and Table 3 for a comprehensive list of medical codes, lab results, and medications utilized.

AKI was assessed over the 10-day post-op period, not inclusive of the surgical day, in the KDIGO-AKI framework with the most severe serum creatinine (SCr) value and presence or absence of RRT. When the patient record had a missing baseline or post-operative SCr reading, the value was imputed according to the following: 1) baseline SCr (measurement closest to index day between – 1 and -90 days; lowest value utilized on closest day if multiple readings available) was imputed to the study mean of the matched cohort; 2) post-operative SCr was imputed to the matched cohort study mean for patients who either did or did not have a diagnosis of acute renal failure (defined as per Supplemental Table 3), depending upon presence of an AKI diagnosis.

Unplanned readmissions within 30 days of index discharge captures acute clinical events that require urgent hospitalization and were determined utilizing the method described by 2015 Measure Updates and Specification report – Hospital-wide All-cause Unplanned Readmissions [2, 3].

**Table S2:** Outcomes definitions and criteria

| **Clinical Outcome** | **Intervention**  **Time frame: Post-Operative day 1 to discharge unless otherwise noted** |
| --- | --- |

**Cardiovascular Outcomes**

| Dysrhythmia | Underwent select procedure [and] received anti-arrhythmics |
| --- | --- |
| SOFA CV Score | SOFA cardiovascular scores[1], scored for all patients |

**Pulmonary Outcomes**

| SOFA Pulmonary | SOFA Pulmonary score [1]  Respiratory support is defined as: ICD-9 procedure codes 96.7x, 31.1, or based  upon ventilator settings present in EHR record |
| --- | --- |
| On-Ventilator  (coded) | Procedure code described as <96 hours duration (through 30-d post discharge)  Procedure code described as >96 hours duration (through 30-d post discharge) |

**Infectious Disease Outcomes**

*All outcomes below require a WBCC*** *>12 x 10^3^/µL* *on same day to +1 day of lab draw [and] antibiotic administered on same day to +3 days of lab draw*

| Confirmed Pneumonia | Positive bronchial lavage (BAL) [or] tracheal aspirate |
| --- | --- |
| Sepsis | Positive blood sample culture |
| Urine infection | Positive urine sample culture |
| Line infection | Positive line sample |

**Renal Outcomes**

***Acute kidney injury (AKI) (Post-operative day 1 – day 10)***

| KDIGO Stage 1 | SCr^‡^ between 1.5 – 2.0x baseline increase [or] ≥ 0.3 mg/dL (≥ 26.5mmol/L) increase |
| --- | --- |
| KDIGO Stage 2 | SCr^‡^ between 2.0 and 2.9-fold baseline increase |
| KDIGO Stage 1 | SCr^‡^ >3.0-fold from baseline increase [or] to ≥ 4 mg/dL (353.6 mmol/L), and initiation of renal replacement therapy |

**Gastrointestinal Outcomes**

| Sequential Organ  Failure Assessment  (SOFA) Liver Score | SOFA Liver Score[1] |
| --- | --- |
| Cholecystitis | Liver Function Result >Upper Normal Limit (UNL)  Alkaline Phosphatase [or] GGT [or] Bilirubin  [and] WBCC** >12 x 10^3^/µL (WBCC on same day to +1 day of liver function test reading) |

* PaO2/FiO2 per lab [or] clinical event numeric values

** WBCC = White Blood Cell Count

‡ Baseline SCr is the numeric value closest in time-proximity prior to cardiac surgery (between -30 to -1 days)

**Table S3:** Summary of medical codes utilized within outcomes definitions

| **ICD-9 or**  **CPT Code** | **Diagnosis or Procedure Description** |
| --- | --- |

**Cardiovascular Outcomes**

***MACE (Major Adverse Cardiac Events): Underwent percutaneous coronary intervention (PCI)***

| 36.01 | Single Vessel Percutaneous Transluminal Coronary Angioplasty or Coronary Atherectomy Without Mention of Thrombolytic Agent |
| --- | --- |
| 36.10 | Single Vessel Percutaneous Transluminal Coronary Angioplasty [PTCA] Or Coronary Atherectomy With Mention of Thrombolytic Agent |
| 36.11 | Open Chest Coronary Artery Angioplasty |
| 36.04 | Intracoronary Artery Thrombolytic Infusion |
| 36.05 | Multiple Vessel Percutaneous Transluminal Coronary Angioplasty [PTCA] Or Coronary Atherectomy Performed During the Same Operation, With or Without Mention of Thrombolytic Agent |
| 36.06 | Insertion of Non-Drug-Eluting Coronary Artery Stent(s) |
| 36.07 | Insertion of Drug-Eluting Coronary Artery Stent(s) |
| 36.09 | Other Specified Removal of Coronary Artery Obstruction |
| 36.2 | Heart Revascularization by Arterial Implant |
| 36.3 | Other Heart Revascularization |
| 36.31 | Open Chest Transmyocardial Revascularization |
| 36.32 | Other Transmyocardial Revascularization |
| 36.33 | Endoscopic Transmyocardial Revascularization |
| 36.39 | Other Heart Revascularization |

***MACE: Stroke / cerebrovascular accident***

| 434  434.0  434.00 | Occlusion of Cerebral Arteries  Cerebral Thrombosis  Cerebral thrombosis without cerebral infarction |
| --- | --- |
| 434.01 | Cerebral thrombosis with cerebral infarction |
| 434.1 | Cerebral embolism |
| 434.10 | Cerebral embolism without mention of cerebral infarction |
| 434.11 | Cerebral embolism with cerebral infarction |
| 434.9  434.90 | Cerebral artery occlusion unspecified, without cerebral infarction  Cerebral artery occlusion, unspecified, with cerebral infarction |
| 434.91 | Cerebral artery occlusion unspecified, with cerebral infarction |
| 435.9  436 | Unspecified transient cerebral ischemia  Acute, but ill-defined, cerebrovascular disease |
| 437.1 | Other generalized ischemic cerebrovascular disease |
| 437.8 | Other ill-defined cerebrovascular disease |
| 437.9 | Unspecified cerebrovascular disease |
| 997.02 | Iatrogenic cerebrovascular infarction or hemorrhage |

***MACE: Myocardial infarction (MI)***

| 410 | Acute MI of anterolateral wall episode of care unspecified |
| --- | --- |
| 410.0 | Acute MI of anterolateral wall episode of care initial episode of care |
| 410.0 | Acute MI of anterolateral wall episode of care subsequent episode of care |
| 410.1 | Acute MI of other anterior wall episode of care unspecified |
| 410.1 | Acute MI of other anterior wall episode of care initial episode of care |
| 410.1 | Acute MI of other anterior wall episode of care subsequent episode of care |
| 410.2 | Acute MI of inferolateral wall episode of care unspecified |
| 410.21 | Acute MI of inferolateral wall initial episode of care |
| 410.22 | Acute MI of inferolateral wall subsequent episode of care |
| 410.3 | Acute MI of inferoposterior wall episode of care unspecified |
| 410.31 | Acute MI of inferoposterior wall initial episode of care |
| 410.32 | Acute MI of inferoposterior wall subsequent episode of care |
| 410.4 | Acute MI of other inferior wall episode of care unspecified |
| 410.41 | Acute MI of other inferior wall initial episode of care |
| 410.42 | Acute MI of other inferior wall subsequent episode of care |
| 410.5 | Acute MI of other lateral wall of care unspecified |
| 410.51 | Acute MI of other lateral wall initial episode of care |
| 410.52 | Acute MI of other lateral wall subsequent episode of care |
| 410.8 | Acute MI of other specified sites episodes of care unspecified |
| 410.81 | Acute MI of other specified sites initial episodes of care |
| 410.82 | Acute MI of other specified sites subsequent episodes of care |
| 410.9 | Acute MI of unspecified site episode of care unspecified |
| 410.91 | Acute MI of unspecified site initial episode of care |
| 410.92 | Acute MI of unspecified site subsequent episode of care |
| 411.81 | Acute coronary occlusion without MI |
| 412 | Old MI |
| 429.71 | Certain sequelae of MI not elsewhere classified acquired cardiac septal defect |
| 429.79 | Certain sequelae of MI not elsewhere classified other |

***Post-surgery cardiac complications***

| 37.41 | Implantation of prosthetic cardiac support device around the heart |
| --- | --- |
| 37.62 | Insertion of non-implantable heart assist system |
| 37.66 | Insertion of implantable heart assist system |
| 37.68 | Insertion of percutaneous external heart assist device |
| 37.82 | Initial insertion of single-chamber device, rate responsive |
| 37.94 | ICD insertion |
| 99.61 | Atrial cardioversion |
| 37.81 | Initial insertion of single-chamber device, not specified as rate responsive |
| 99.62 | Other electric countershock of heart |

***New-Onset heart failure***

| 428 | Congestive heart failure, unspecified |
| --- | --- |
| 428.1 | Left heart failure |
| 428.2 | Unspecified systolic heart failure |
| 428.3 | Unspecified diastolic heart failure |
| 428.31 | Acute diastolic heart failure |
| 428.33 | Acute on chronic diastolic heart failure |
| 428.4 | Unspecified combined systolic and diastolic heart failure |
| 428.41 | Acute combined systolic and diastolic heart failure |
| 428.43 | Acute on chronic combined systolic and diastolic heart failure |
| 428.9 | Heart failure unspecified |
| 428.21 | Acute systolic heart failure |
| 428.23 | Acute on chronic systolic heart failure |

**Diagnosed Respiratory Failure**

| 518.4 | Acute edema of lung, unspecified |
| --- | --- |
| 518.5 | Pulmonary insufficiency following trauma and surgery |
| 518.51 | Acute respiratory failure following trauma and surgery |
| 518.52 | Other pulmonary insufficiency, not elsewhere classified, following trauma and surgery |
| 518.53 | Acute and chronic respiratory failure following trauma and surgery |
| 518.81 | Acute respiratory failure |
| 518.82 | Other pulmonary insufficiency, not elsewhere classified |
| 96.04 | Insertion of Endotracheal Tube |
| 96.70 | Continuous mechanical ventilation of unspecified duration |
| 96.71 | Continuous invasive mechanical ventilation less than 96 consecutive hrs |
| 96.72 | Continuous invasive mechanical ventilation for 96 consecutive hrs or more |

**Any Diagnosed Infection**

| 038.xx | Septicemia |
| --- | --- |
| 790.7 | Bacteremia |
| 041.xx | Bacterial infections unspecified site |
| 997.31 | Ventilator associated pneumonia |
| 481 | Pneumococcal pneumonia [Streptococcus pneumoniae pneumonia] |
| 482.41 | Methicillin susceptible pneumonia due to Staphylococcus aureus |
| 482.8x | Pneumonia due to other specified bacteria |
| 484.xx | Pneumonia in infectious diseases classified elsewhere |
| 486 | Pneumonia, organism unspecified |
| 510.xx | Empyema |
| 567.22 | Peritoneal abscess |
| 567.29 | Other suppurative peritonitis |
| 569.5x | Abscess of intestine |
| 519.2 | Mediastinitis |
| 995.91 | Sepsis |
| 995.92 | Severe sepsis |
| 998.3x | Disruption of wound/dehiscence |
| 999.31 | Other and unspecified infection due to central venous catheter |
| 999.32 | Bloodstream infection due to central venous catheter |
| 999.33 | Local infection due to central venous catheter |
| 86.22 | Excisional debridement of wound, infection, or burn |
| 86.28 | Nonexcisional debridement of wound, infection or burn |

**Bleeding Outcomes**

***Hemorrhage***

| 287.9 | Unspecified hemorrhagic conditions |
| --- | --- |
| 459 | Hemorrhage, unspecified |
| 958.2 | Secondary and recurrent hemorrhage |
| 998.1 | Hemorrhage or Hematoma Complicating a Procedure |
| 998.11 | Hemorrhage complicating a procedure |
| 998.12 | Hematoma complicating a procedure |

***Transfusion***

| 99.02 | Transfusion of previously collected autologous blood |
| --- | --- |
| 99.03 | Other transfusion of whole blood |
| 99.04 | Transfusion of packed cells |
| 99.05 | Transfusion of platelets |
| 99.06 | coagulation factors |
| 99.07 | Other serum |
| 99.09 | Transfusion of other substance |
| 99.0 | Transfusion of Blood and Blood Components |

**Acute Renal Failure**

| 39.95 | Dialysis |
| --- | --- |
| 38.95 | Venous catheter for renal dialysis |
| 584.xx | Acute renal failure |
| 586 | Renal failure, unspecified |
| 588.8 | Other specified disorders resulting from impaired renal function |
| 588.89 | Other specified disorders resulting from impaired renal function -Impair renal function dis NEC |
| 588.9 | Unspecified disorder resulting from impaired renal function |
| 90935 | Hemodialysis procedure with single evaluation by a physician or other qualified health care professional |
| 90937 | Hemodialysis procedure requiring repeated evaluation(s) with or without substantial revision of dialysis prescription |
| 90945 | Dialysis procedure other than hemodialysis (eg, peritoneal dialysis, hemofiltration, or other continuous renal replacement therapies), with single evaluation by a physician or other qualified health care professional |
| 90947 | Dialysis procedure other than hemodialysis (eg, peritoneal dialysis, hemofiltration, or other continuous renal replacement therapies) requiring repeated evaluation by a physician or other qualified health care professional, with or without substantial rev |

**Neurologic Complications**

| 292.81 | Drug-induced delirium |
| --- | --- |
| 293.0 | Delirium due to conditions classified elsewhere |
| 293.1 | Subacute delirium |
| 293.89 | Other specified transient mental disorders due to conditions classified elsewhere, other |
| 293.9 | Unspecified transient mental disorder in conditions classified elsewhere |
| 431 | Intracerebral hemorrhage |
| 434.1x | Cerebral embolism |
| 434.9x | Cerebral artery occlusion, unspecified |

**Renal Replacement Therapy**

| 38.95 | Venous catheterization for renal dialysis |
| --- | --- |
| 39.95 | Hemodialysis |
| 90935 | Hemodialysis procedure w/1 physician evaluation |
| 90937 | Hemodialysis procedure requiring repeated evaluation(s) with or without substantial revision of dialysis prescription |
| 90947 | Inpatient visits for dialysis procedures other than hemodialysis (e.g., peritoneal dialysis, hemofiltration, or continuous renal replacement therapies) |

**Gastrointestinal Complications**

| 530.12 | Acute esophagitis |
| --- | --- |
| 535.xx | Gastritis and duodentitis  Excluding: 535.3 – Alcoholic Gastritis 535.31 - Alcoholic Gastritis, without Mention of Hemorrhage 535.32 - Alcoholic Gastritis, with Hemorrhage |
| 557 | Acute vascular insufficiency of intestine |
| 557.9 | Unspecified vascular insufficiency of intestine |
| 574.0x | Calculus of gallbladder with acute cholecystitis |
| 574.3x | Calculus of bile duct with acute cholecystitis |
| 574.0x | Calculus of gallbladder with acute cholecystitis |
| 575 | Acute cholecystitis |
| 578.xx | Gastrointestinal bleed |
| 531.0x | Acute gastric ulcer with hemorrhage |

**Figure S4a.** Cardiopulmonary outcomes in matched cohort of patients with PAC and without PAC one day post-cardiac surgery through 30 days post-discharge

[Dysrhythmia calculated one day post-cardiac surgery through discharge]

**
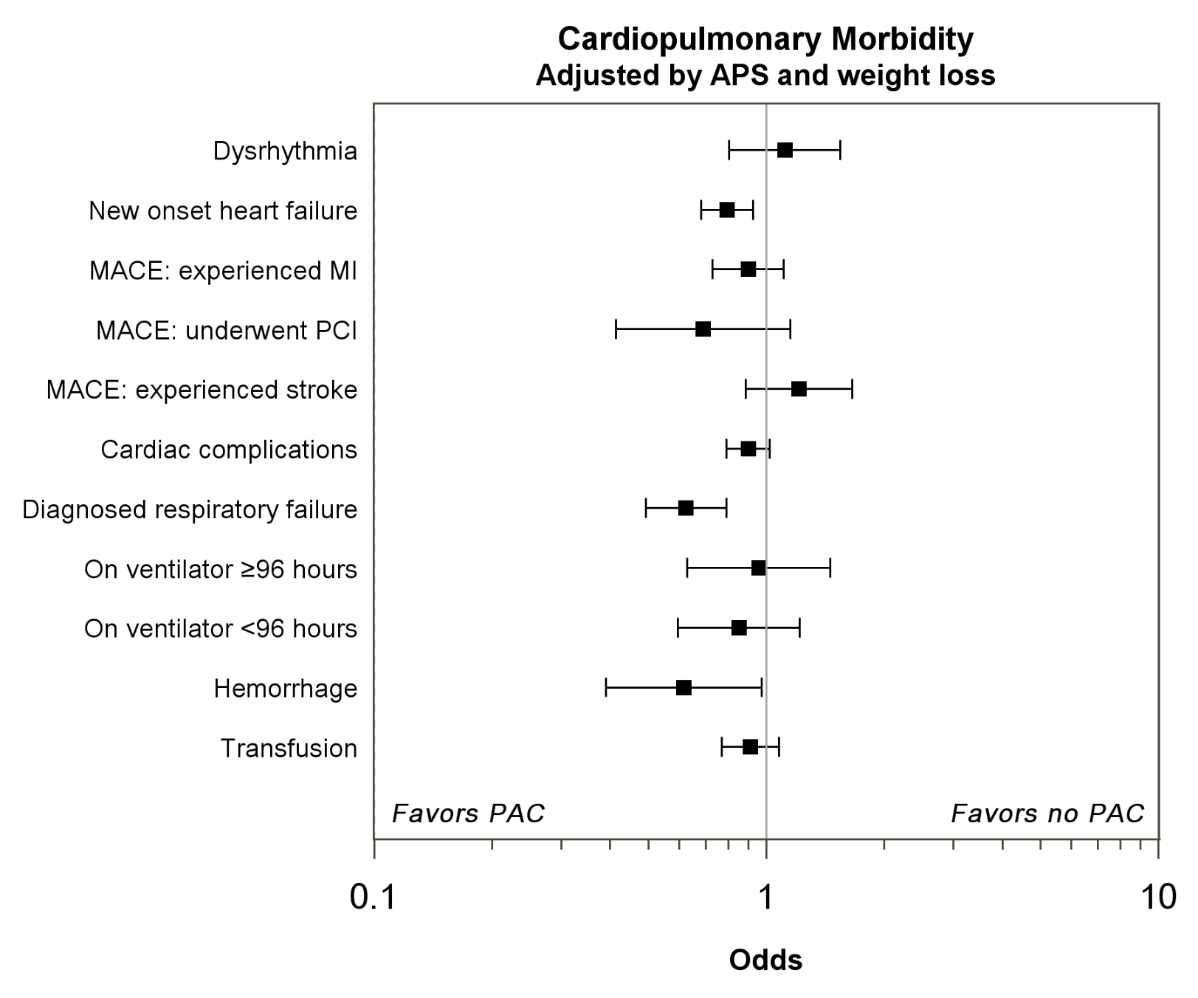
**

**Figure S4b.** Infectious morbidity in matched cohort of patients with PAC and without PAC one day post-cardiac surgery through discharge

[Any diagnosed infection calculated one day post-cardiac surgery through 30 days post-discharge]

**Figure S4c.** Other exploratory outcomes in matched cohort of patients with PAC and without PAC.

[Time frames for outcomes are post-operative day one through: discharge (renal replacement therapy, cholecystitis, SOFA CV abnormal); Day 10 (post-op KDIGO AKI); 30 days post discharge (acute renal failure, gastrointestinal complication, neurologic complication, readmissions)]

**
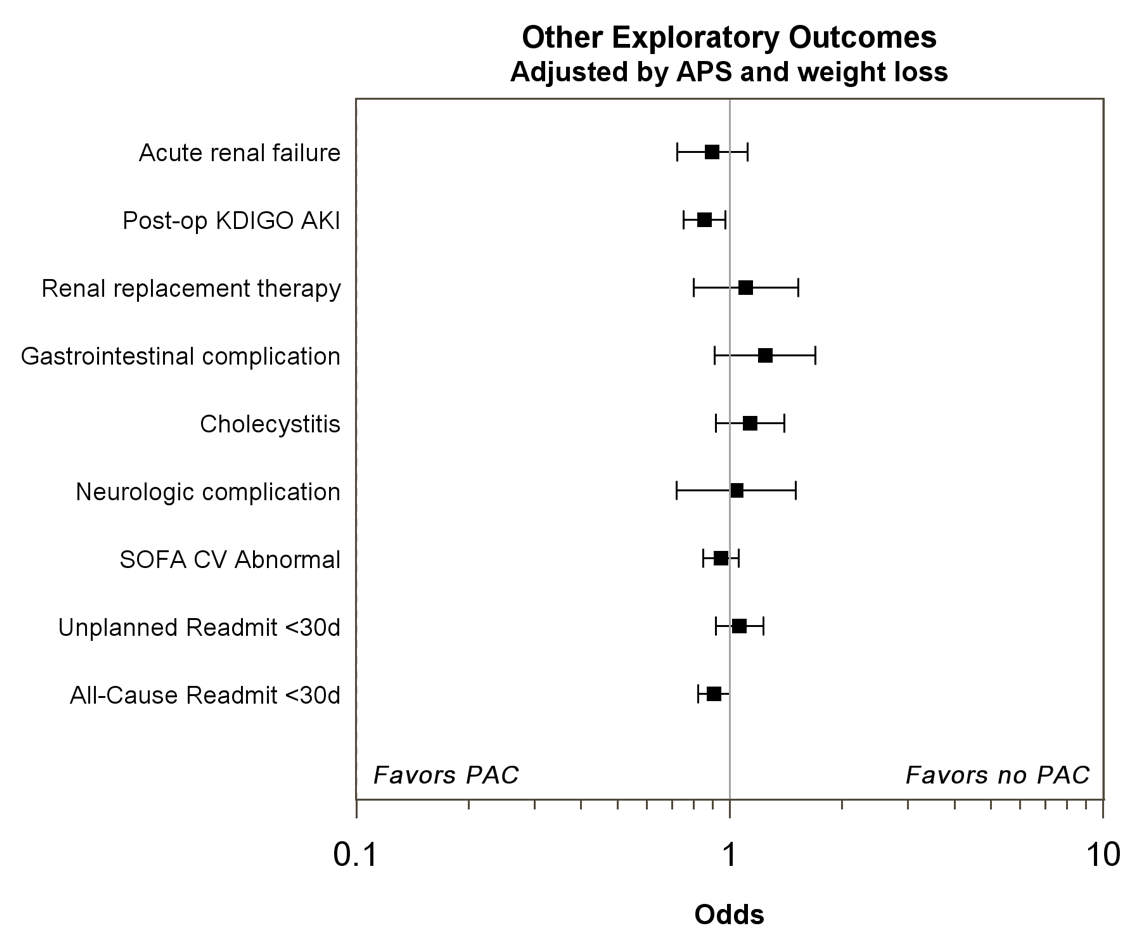
Table S5a:** Primary outcomes frequencies and models statistics

| **Primary Outcomes** | **Adjusted Outcomes** | | | | **Unadjusted Outcomes** | | | |
| --- | --- | --- | --- | --- | --- | --- | --- | --- |
|  | **Odds ratio** | **Lower**  **Cl** | **Upper**  **Cl** | ***p*-value** | **Odds ratio** | **Lower**  **Cl** | **Upper**  **Cl** | ***p*-value** |
| In-hospital mortality | 1.165 | 0.646 | 2.101 | 0.516 | 1.092 | 0.611 | 1.950 | 0.706 |
| Cardiopulmonary morbidity | 0.871 | 0.788 | 0.963 | <0.001 | 0.874 | 0.791 | 0.965 | <0.001 |
| Infectious morbidity | 1.280 | 1.100 | 1.490 | <0.001 | 1.254 | 1.080 | 1.457 | <0.001 |

**Table S5b:** Cardiopulmonary outcome frequencies and models statistics

| **Cardiopulmonary** | **Adjusted Outcomes** | | | | **Unadjusted Outcomes** | | | |
| --- | --- | --- | --- | --- | --- | --- | --- | --- |
| **Outcomes** | **Odds ratio** | **Lower**  **Cl** | **Upper**  **Cl** | ***p*-value** | **Odds ratio** | **Lower**  **Cl** | **Upper**  **Cl** | ***p*-value** |
| **Cardiac Outcomes** |  |  |  |  |  |  |  |  |
| Dysrhythmia | 1.115 | 0.805 | 1.545 | 0.513 | 1.101 | 0.795 | 1.524 | 0.562 |
| New onset heart failure | 0.794 | 0.681 | 0.926 | 0.003 | 0.797 | 0.684 | 0.929 | 0.003 |
| MACE: experienced MI | 0.899 | 0.728 | 1.109 | 0.320 | 0.897 | 0.727 | 1.106 | 0.309 |
| MACE: underwent PCI | 0.690 | 0.414 | 1.150 | 0.154 | 0.673 | 0.405 | 1.121 | 0.128 |
| MACE: experienced stroke | 1.212 | 0.887 | 1.657 | 0.227 | 1.208 | 0.884 | 1.651 | 0.235 |
| Cardiac complications | 0.899 | 0.791 | 1.021 | 0.101 | 0.904 | 0.795 | 1.026 | 0.118 |

**Respiratory Outcomes**

| Diagnosed respiratory failure | 0.624 | 0.493 | 0.791 | <0.001 | 0.618 | 0.488 | 0.782 | <0.001 |
| --- | --- | --- | --- | --- | --- | --- | --- | --- |
| On ventilator >96 hours | 0.958 | 0.630 | 1.456 | 0.839 | 0.897 | 0.595 | 1.351 | 0.601 |
| On ventilator <96 hours | 0.852 | 0.596 | 1.218 | 0.379 | 0.823 | 0.578 | 1.173 | 0.281 |

**Bleeding / hematologic Outcomes**

| Hemorrhage | 0.616 | 0.390 | 0.974 | 0.038 | 0.609 | 0.386 | 0.961 | 0.033 |
| --- | --- | --- | --- | --- | --- | --- | --- | --- |
| Transfusion | 0.911 | 0.769 | 1.080 | 0.283 | 0.905 | 0.765 | 1.071 | 0.246 |

**Table S5c:** Infectious morbidity frequencies and models statistics

| **Infectious morbidity** | **Adjusted Outcomes** | | | | **Unadjusted Outcomes** | | | |
| --- | --- | --- | --- | --- | --- | --- | --- | --- |
|  | **Odds ratio** | **Lower**  **Cl** | **Upper**  **Cl** | ***p*-value** | **Odds ratio** | **Lower**  **Cl** | **Upper**  **Cl** | ***p*-value** |
| Confirmed pneumonia | 1.097 | 0.683 | 1.763 | 0.700 | 1.059 | 0.661 | 1.697 | 0.810 |
| Bacteremia | 1.362 | 1.020 | 1.819 | 0.036 | 1.316 | 0.988 | 1.753 | 0.060 |
| Urine infection | 1.577 | 1.206 | 2.061 | <0.001 | 1.555 | 1.191 | 2.032 | 0.001 |
| Line infection | 0.982 | 0.412 | 2.337 | 0.966 | 0.909 | 0.385 | 2.143 | 0.827 |
| Any diagnosed infection | 1.191 | 0.980 | 1.448 | 0.078 | 1.182 | 0.973 | 1.435 | 0.092 |

**Table S5d:** Other exploratory outcomes frequencies and models statistics

| **Other Exploratory Outcomes** | **Adjusted Outcomes** | | | | **Unadjusted Outcomes** | | | |
| --- | --- | --- | --- | --- | --- | --- | --- | --- |
|  | **Odds ratio** | **Lower**  **Cl** | **Upper**  **Cl** | ***p*-value** | **Odds ratio** | **Lower**  **Cl** | **Upper**  **Cl** | ***p*-value** |

**Renal Outcomes**

| Acute renal failure | 0.898 | 0.723 | 1.114 | 0.327 | 0.892 | 0.719 | 1.106 | 0.296 |
| --- | --- | --- | --- | --- | --- | --- | --- | --- |
| Post-op KDIGO AKI | 0.856 | 0.753 | 0.972 | 0.016 | 0.851 | 0.750 | 0.966 | 0.012 |
| Renal replacement therapy | 1.105 | 0.802 | 1.523 | 0.542 | 1.039 | 0.760 | 1.420 | 0.810 |

**Gastrointestinal Outcomes**

| Gastrointestinal complications | 1.243 | 0.911 | 1.696 | 0.170 | 1.236 | 0.906 | 1.686 | 0.181 |
| --- | --- | --- | --- | --- | --- | --- | --- | --- |

**Liver Outcomes**

| Cholecystitis | 1.133 | 0.918 | 1.399 | 0.243 | 1.085 | 0.884 | 1.332 | 0.433 |
| --- | --- | --- | --- | --- | --- | --- | --- | --- |

**Neurologic Outcomes**

| Neurologic complication | 1.042 | 0.721 | 1.504 | 0.827 | 1.036 | 0.717 | 1.495 | 0.851 |
| --- | --- | --- | --- | --- | --- | --- | --- | --- |
| SOFA CV Abnormal | 0.947 | 0.848 | 1.058 | 0.338 | 0.889 | 0.800 | 0.988 | 0.028 |
| **Readmissions** |  |  |  |  |  |  |  |  |
| Unplanned Readmissions <30d | 1.063 | 0.917 | 1.231 | 0.418 | 1.067 | 0.921 | 1.236 | 0.388 |
| All-Cause Readmissions <30d | 0.908 | 0.824 | 1.000 | 0.050 | 0.917 | 0.833 | 1.010 | 0.077 |

**Information S2:** EuroSCORE II evaluation

EuroScore II was calculated following the manuscript by Nashef SA et al.[4] with minor modification based on the data available within Cerner Health Facts^®^. Disease and operation related factors were defined using ICD-9 and CPT-4 codes, medication use, and lab results (for detailed definitions, see supplemental data). Parameters used in the modified EuroScore II calculation were the same as the original manuscript (Nashef SA et al., 2012) [4]. All analyses were carried out using SAS^®^9.4.

**Definitions of risk factors in EuroScore II calculation are as follows:**

***Renal impairment:***

Same as the original paper. On dialysis was defined by presence of diagnosis or procedure codes indicating that patients were on dialysis (39.95, 54.98, 39.93, 39.43, 39.42, 39.27, V56.8, 996.56, 996.68, 996.73, E870.2, E871.2, E872.2, E874.2, E879.1, V45.1, V56, V56.0 to V56.2, 792.5, 458.21, V45.11) within one year prior to the date of qualifying cardiac procedure. For creatinine clearance, results from most recent date were chosen if there were measures from multiple days.

***Extracardiac arteriopathy:***

Defined by presence of diagnosis or procedure codes indicating that patients had extracardiac arteriopathy (35471, 35475, 37220, 37222, 37224, 37228, 37232, 37221, 37223, 37226, 37230, 37234, 37236, 37237, 00.61- 00.65, 00.44, 99.10, 00.45-00.48, 00.40-00.43, 39.50, 00.55, 00.60, 39.90, 39.29) any time prior to the date of qualifying cardiac procedure.

***Poor mobility:***

Defined by presence of ICD-9 diagnosis codes 358.xx, 359.xx, 342.xx, 343.xx, 344.xx, or 332.xx any time prior to the date of qualifying cardiac procedure.

***Previous cardiac surgery:***

Based on the inclusion criteria of this study, none of the patients had a previous major cardiac operation involving opening the pericardium.

***Chronic lung disease:***

Defined by presence of ICD-9 diagnosis codes 490-492.8, 493.00-493.92, 494-494.1, 495.0-505, or 506.4 any time prior to the date of qualifying cardiac procedure.

***Active endocarditis:***

Defined by presence of ICD-9 diagnosis codes 421.0 or 421.1, one to thirty days prior to cardiac procedure date.

***Critical preoperative state:***

Defined by Inotropes (dobutamine, dopamine, isoprinosine, epinephrine, norepinephrine, milrinone, phenylephrine) use the day prior to the date of qualifying cardiac procedure.

***Diabetes on insulin:***

Defined by presence of ICD-9 code 250.01 or use of insulin therapy any time prior to the date of qualifying cardiac procedure.

***NYHA:***

NYHA stage is not captured within the Cerner Health Facts^®^ database. This was defined by congestive heart failure (CHF) ICD-9 diagnosis codes 428.0, 428.1, 428.20-428.23, 428.30 to 428.33, 428.40-428.43 any time prior to the date of qualifying cardiac procedure, and/or administration of diuretics, angiotensin converting enzyme inhibitors (ACE inhibitors) / angiotensin Receptor Blockers (ARBs), or carvedilol within one year prior to the date of qualifying cardiac procedure.

- Stage I: no CHF diagnosis code present and none of the three medication classes were administered;
- Stage II: presence of CHF diagnosis codes or a single class of medication was administered;
- Stage III: two classes of medication were administered;
- Stage IV: three or more classes of medication were administered.

***CCS class 4 angina:***

Defined by presence of ICD-9 diagnosis code 411.1 any time prior to the date of qualifying cardiac procedure.

***LV function:***

Defined by clinical LVEV measurements within one to ninety days prior to the date of qualifying cardiac procedure. If multiple measurement day results were available, the result from the date closest to the date of qualifying cardiac procedure was used. If multiple measurements were available for the same day, the least severe measurement was used.

***Recent MI:***

Defined by presence of ICD-9 diagnosis codes 410.xx present one to ninety days prior to the date of qualifying cardiac procedure.

***Pulmonary hypertension:***

Defined by presence of ICD-9 diagnosis codes 416.0 or 416.8 (assumed 'Moderate' if present; otherwise considered 'None') one to ninety days prior to the date of qualifying cardiac procedure.

***Urgency:***

Defined by patients’ admission type (admission type = elective as “elective”, admission type = urgent as “urgent”, admission type = emergency as “emergency”). The “salvage” category was not utilized due to the inability to determine whether external cardiac massage was implemented prior to induction of anesthesia within the Cerner Health Facts^®^ database. Patient records without an admission type were defined as “emergency”.

***Weight of the intervention:***

Defined by ICD-9 or CPT-4 procedure codes for the index procedures. The baseline intervention is isolated CABG. Operations more complex than baseline fall into three categories with increasing weight:

- Isolated non-CABG major procedure (only 1 of the following: isolated valve, heart transplant, PCI, other vascular, aortic procedure)
- Two major procedures (any 2 of the following, in any combination: CABG, valve, heart transplant, PCI, other vascular, aortic procedure)
- Three or more major procedures (any 3 or more of the following, in any combination: CABG, valve, heart transplant, PCI, other vascular, aortic procedure)

(Please see supplemental excel file for codes for each procedure categories)

***Surgery on thoracic aorta:***

Defined by presence of relevant ICD-9 or CPT-4 codes (39.73, 33875, 33880, 33881, 33883, 33884, 33886, 33889, 33891, 75956 to 75959, 36221) for index procedures combined with an ICD-9 procedure code of 39.61 (Extracorporeal circulation auxiliary to open heart surgery) or a procedure code whose title includes cardiopulmonary bypass (CPB).

**Table S6:** Parameters to determine the patient predicted mortality rate by modified EuroSCORE II

| **Parameter** | **Categories** | **Description** |
| --- | --- | --- |
| Age | ≤60 of >60 | Age of patient; limited data was available for patients >90 years old |
| Female | Yes or No | Patient gender |
| Renal impairment | On dialysis, moderately impaired renal function (50-85 ml/min) or severely impaired renal function (<50 ml/min) not on dialysis | Calculated creatine clearance times were utilized for group assignment that incorporated age, weight and serum creatine  Timeframe: 1 day to 1 year prior to qualifying cardiac surgery |
| Extracardiac arteriopathy | Yes or No | Includes amputation for arterial disease, claudication, carotid occlusion or >50% stenosis, and intervention on the abdominal aorta, limb arteries or carotids  Timeframe: Any time prior to surgery |
| Poor mobility | Yes or No | Musculoskeletal or neurological dysfunction that leads to severe mobility impairment*  Timeframe: At least one day prior to procedure |
| Chronic lung disease | Yes or No | Chronic pulmonary dysfunction was considered (utilized Elixhauser comorbidity codes) |
| Active endocarditis | Yes or No | Utilized ICD-9 codes 421.0 Acute and subacute bacterial endocarditis and 421.1 Acute and subacute infective endocarditis in diseases classified elsewhere  Timeline: 1 – 30 days prior to qualifying cardiac surgery |
| Critical preoperative state | Yes or No | Inotropes (dobutamine, dopamine, isoprenaline, epinephrine, norepinephrine, milrinone, phenylephrine) received the day prior to qualifying cardiac procedure  Timeframe: Day prior to surgery |
| Diabetes on insulin | Yes or No | This parameter included Type I diabetes, diabetes mellitus without complication, not considered uncontrolled, or any prior use of insulin therapy up to the date prior to cardiac surgery* |
| NYHA (New York Heart Association) | Four classes for dyspnea:  I: no symptoms on moderate exertion  II: symptoms on moderate exertion  III: symptoms on light exertion  IV: symptoms at rest | Health Facts does not include NYHA class. Utilized medications received of three classes to assign the NYSE classification (Class 1 - Diuretics; Class 2: Angiotensin Receptor Blockers and ACE Inhibitors; Class 3 Carvedilol (Coreg) single medication [not including other beta blockers]).  Assigned a NYSE class II if patient received one medication class; NYSE class III if received two classes of medication; and NYSE class IV if patient received three classes of medication  Timeframe: medications in the 90 days prior to cardiac procedure |
| CCS class 4 angina | Yes or No | ICD9 code 411.1 (Intermediate coronary syndrome) utilized to signify class 4 angina  Timeframe: Any diagnosis prior to index surgery |
| LV function | Very poor (LVEF: 20% or less)  Poor (LVEF: 21% - 30%)  Moderate (LVEF: 31 – 50%)  Good (LVEF: 51% or more) | LVEF categories were assigned based upon feasibility from Cerner LVEF codes (very poor, poor, moderate) if present; “Good” was not assigned  Timeframe: 1 to 90 days preop, using the most recent date if measures for multiple days were present |
| Recent MI | Yes or No | Included if patient experienced a myocardial infarction  Timeframe: 90 days prior to cardiac procedure |
| Pulmonary hypertension | None  Moderate (PA systolic pressure 31-55 mmHg)  Severe (PA systolic pressure greater than 55 mmHg) | Utilized ICD-9 diagnosis codes 416.0 Primary pulmonary hypertension and 416.8 Other chronic pulmonary heart diseases; presence of diagnosis codes defined moderate hypertension. Severe condition designation was not included.  Timeframe: Any prior diagnosis, including the index visit if the diagnosis was present upon admission or the reason for the visit |
| Urgency | Elective (routine admission for procedure); urgent (patient not electively admitted and requires procedure before discharge); emergency (operation occurs within a day of the decision to operate) or salvage (patient requires cardiopulmonary resuscitation prior to induction of anesthesia or en route to operating room) | Elective, urgent, and emergency admissions were identified as represented in the electronic database. Salvage patients would be included within the “Emergency” group (cannot be discerned within Cerner) |
| Weight of Intervention | Isolated CABG, single non-CABG, two major procedures, three or more major procedures | Procedures considered include valves, bypass, and aorta surgeries, etc., but did not include procedures involving sternotomy, insertion of intra-aortic balloon, closure procedures, etc. |
| Surgery on thoracic aorta | Yes or No | Utilized ICD-9 and CPT-4 procedure codes* for the thoracic aorta |

**REFERENCES**

1. Vincent JL, Moreno R, Takala J, Willatts S, De Mendonca A, Bruining H, Reinhart CK, Suter PM, Thijs LG. The SOFA (Sepsis-related Organ Failure Assessment) score to describe organ dysfunction/failure. On behalf of the Working Group on Sepsis-Related Problems of the European Society of Intensive Care Medicine. Intensive Care Med 1996; 22(7):707-10.

2. 2015 Measure Updates and Specifications Report

Hospital-Wide All-Cause Unplanned Readmission Measure – Version 4.0. Centers for Medicare & Medicaid Services 2015.

3. Horwitz LI, Partovian C, Lin Z, Grady JN, Herrin J, Conover M, Montague J, Dillaway C, Bartczak K, Suter LG *et al*. Development and use of an administrative claims measure for profiling hospital-wide performance on 30-day unplanned readmission. Annals of internal medicine 2014; 161(10 Suppl):S66-75.

4. Nashef SA, Roques F, Sharples LD, Nilsson J, Smith C, Goldstone AR, Lockowandt U. EuroSCORE II. Eur J Cardiothorac Surg 2012; 41(4):734-744; discussion 744-35.
